# Large-Scale Evidence for Conservation of NMD Candidature Across Mammals

**DOI:** 10.1371/journal.pone.0011695

**Published:** 2010-07-21

**Authors:** David A. de Lima Morais, Paul M. Harrison

**Affiliations:** Department of Biology, McGill University, Montreal, Quebec, Canada; University of California Riverside, United States of America

## Abstract

**Background:**

Alternatively-spliced (AS) forms can vary protein function, intracellular localization and post-translational modifications. AS coupled with mRNA nonsense-mediated decay (NMD) can also control the transcript abundance. Here, we have investigated the genome-scale conservation of alternatively-spliced NMD candidates (AS-NMD candidates), in mammals.

**Methodology/Principal Findings:**

We mapped >12 million cDNA/EST library transcripts, comprising pooled data from both older and next-generation sequencing techniques, against genomic sequences to annotate AS-NMD candidates generated by in-frame premature termination codons (PTCs), in the human, mouse, rat and cow genomes. In these genomes, we found populations of genes that harbour AS-NMD candidates, varying in number from ∼149 to 2,051 genes. We discovered that a highly-significant proportion (27%–35%) of AS-NMD candidate genes in mouse, rat and cow, also have human orthologs targeted for NMD. Intron retention was the most abundant type of AS-NMD, ranging from 43% to 67% of genes harbouring an AS-NMD candidate. Groupings of AS-NMD candidate genes either with or without intron retentions also have highly significant AS-NMD conservation, indicating that the trend is not due primarily to conservation of intron retentions. As a subset, the AS-NMD intron retentions are distinguished from non-retained introns by higher GC content, and codon usage similar to the usage in protein-coding sequences. This indicates that most of these alternatively spliced sequences have coded for proteins in the recent evolutionary past. In general, the AS-NMD candidate genes showed a similar pattern of Gene Ontology functional category enrichments in all four species. Genes linked to nucleic-acid interaction and apoptosis, and involved in pathways linked with cancer, were the most common. Finally, we mapped the AS-NMD candidates to mass spectrometry-derived proteomics data, and gathered evidence of truncated polypeptides for at least 10% of all human AS-NMD candidate transcripts.

**Conclusions/Significance:**

In summary, our analysis provides strong statistical evidence for conservation of functional AS-NMD candidature across *Mammalia* for a large subset of genes. However, because codon usage of AS-NMD intron retentions is similar to the usage in exons, it is difficult to de-couple conservation of AS-NMD-based regulation from conservation for protein-coding ability, for intron retentions.

## Introduction

Alternative splicing is the generation of multiple transcripts from a single protein-coding gene. Several studies have shown that at least half of all human genes undergo alternative splicing [1], [2], although RNA array and sequencing data shows that this number is likely as high as 94% of genes [3]. Alternatively-spliced (AS) forms can vary protein function, intracellular localization and post-translational modifications. Although, in most cases, protein AS isoforms show only small differences, some isoforms can lead to large functional variances and even to genetic disorders [4]. Apparently, alternative splicing can also control transcript abundance, due to its coupling to mRNA nonsense-mediated decay (NMD). It seems that at least 10–15% of human transcripts can be switched off by NMD coupled to alternative splicing [5].

Nonsense-mediated decay is a eukaryotic surveillance mechanism that detects mRNAs that harbour premature termination codons (PTCs), and commits these transcripts to rapid decay. One of the expected physiological consequences of NMD is the prevention of synthesis of truncated polypeptides, which presumably leads to protection of the cell from resultant deleterious dominant-negative or gain-of-function effects [6], [7]. The biological importance of NMD is highlighted by the fact that 30% of inherited genetic diseases are due to PTC, most of which are in NMD targets [8]. Therefore, the majority of the nonsense-associated diseases are caused either by an insufficient level of the functional protein as a result of degradation of a PTC-containing mRNA, or by generation of a defective truncated protein from a PTC-containing mRNA that escaped the NMD surveillance [4], [8].

In mammalian cells, translation termination codons and exon–exon junctions are *cis*-acting elements that allow recognition of PTCs. The mRNA is subject to rapid decay when there is a PTC more than ∼50–55 nucleotides upstream of the last exon–exon junction [8]. The positional information that marks exon–exon junctions is provided by the components of the splicing-dependent exon junction complex (EJC) that persist during export, and until the mRNA is translated [9]. NMD is believed to require three core UPF factors which are conserved from yeast to humans, namely, UPF1 (also known as regulator of nonsense transcripts 1 [RENT1]), UPF2 (RENT2), and UPF3 [10]. UPF1 is not only essential to NMD but is also required for rapid degradation of histone mRNAs [11].

Here, we have focused on the analysis of orthologous alternatively-spliced NMD candidates (AS-NMD candidates). We have annotated and analyzed the occurrence and conservation of AS-NMD candidates in genes, generated by in-frame premature stop codons, in four mammalian genomes: human, mouse, rat and cow. Strikingly, we find highly significant conservation of NMD candidature, and also demonstrate that genes containing an AS-NMD candidate are significantly associated with specific functional categories of proteins, and of disease-associated genes.

## Results and Discussion

### AS-NMD annotation overview

To assess the existence in public databases of alternatively-spliced forms that are putative candidates for nonsense-mediated mRNA decay (hereafter termed ‘AS-NMD candidates’), we developed and applied a pipeline to four mammalian genomes (human, mouse, rat and cow). This pipeline is described in detail, in *Methods*. We annotated AS-NMD candidates as transcripts containing in-frame premature termination codons (PTCs) >50 nucleotides 5′ of the last exon splice junction. In brief, we mapped the complete corpus of >12 million cDNA/EST transcript library sequences from several databases (RefSeq, Unigene, dbEST and H-invitational) against genomic sequences for the four mammals (human, mouse, rat and cow). This data set contains pooled transcript data both from older sequencing techniques and from next-generation sequencing methods. We compared these complete transcript library mappings to mappings to reference mRNA mappings for each gene, to calculate whether the mapped expressed sequence is part of an AS-NMD candidate.

The pipeline identified between ∼149 and ∼2051 genes with AS-NMD candidates, per genome (Table 1). The number of supporting cDNAs/ESTs varied between just 190 in rat, to 14,343 in human. The proportions of genes with AS-NMD candidates are between 1% and 10% of genes in each genome. However, it is important to be aware that, as for any analysis of transcript data, the major difference between several studies lies in the number of transcript sequences considered. For example, Zhang *et al.* (2009 [12]), reported that, the number of predicted AS-NMD candidates increased when these authors included predicted RefSeq ESTs in their analysis.

**Table 1:** Summary of the AS-NMD candidates.

Genomes	(1) AS events	(2) EST/cDNA transcripts supporting the AS	(3) Genes with an AS-NMD candidate	(4) Number of (1) that are *only* in the Refseq or Unigene databases	(5) Proportion of (3) that have orthologs* in the human genome	(6) AS-NMD candidate genes with an orthologous AS-NMD candidate in the human genome*
*Homo sapiens*	2536	14343	2051 (9.8%)†	12/2536 (0.5%)	--------	--------
*Mus musculus*	1211	1628	1069 (4.6%)†	23/1211 (1.9%)	970/1069 (91%)	262/970 (27%) ∇
*Rattus norvegicus*	166	190	149 (0.84%)†	6/166 (3.6%)	129/149 (87%)	45/129 (34%) ∇
*Bos taurus*	374	600	342 (1.8%)†	4/374 (1.1%)	297/342 (87%)	105/297 (35%) ∇

† Percentage of the total number of known genes based on Ensembl build 52.

†† Percentage of retained introns whose length is a multiple of 3.

* Orthology was based on the relationship one-to-one between a human transcript and a transcript from the other genome according to Ensembl annotation.

∇Significant by a hypergeometric probability test with P ≤10^−10^. We calculated the probability of picking the observed number of AS-NMD candidates in the other mammals that are conserved in human, from the total population of human genes.

We classified the AS-NMD candidates according to the type of events that they present: IR, intron retention; CSE, cassette exon; ASD, alternative splicing donor; ASA, alternative splicing acceptor (Tables 2–3). Surprisingly, IR was the most abundant event in all genomes. It varied from 43% to 67% of all genes that harbour AS-NMD candidates. ASA was the second most abundant event (19%–32%). Due to the large number of IRs, we present below a set of analyses to verify whether or not—as a population—the IRs are artifacts derived from unprocessed or partially processed pre-mRNAs.

**Table 2:** Summary of the AS-NMD candidates involving intron retentions (IRs).

Genomes	(1) EST/cDNA transcripts supporting the IR	(2) Genes with an AS-NMD candidate involving an IR	(3) IRs with lengths that are multiples of 3*n* ††	(4) Orthologs* in the human genome	(5) AS-NMD candidate genes with an orthologous AS-NMD candidate in the human genome*	(6) Proportion of cases in (5) that are in the same position in the gene †††
*Homo sapiens*	2117	884	37%	--------	--------	--------
*Mus musculus*	725	475	28%	440	153/475 (35%) ∇∇∇	20/153 (13%)
*Rattus norvegicus*	123	95	36%	92	32/95 (35%) ∇	7/32 (22%)
*Bos taurus*	428	231	30%	211	85/231 (40%) ∇∇	17/85 (20%)

† Percentage of the total number of known genes based on Ensembl build 52.

†† Percentage of retained introns whose length is a multiple of 3.

††† The percentage is based on the number of AS-NMD candidates with an orthologous AS-NMD candidates in the human genome (Table 1).

* Orthology was based on the relationship one-to-one between a human transcript and a transcript from the other genome according to Ensembl annotation.

∇ Significant by a hypergeometric probability test with P ≤10^−10^. We calculated the probability of picking the observed number of AS-NMD candidates in the other mammals that are conserved in human, from the total population of human genes.

**Table 3:** Number of events per genome.

Events	*Homo sapiens*	*Mus musculus*	*Rattus norvegicus*	*Bos taurus*
IR†	884 (43%)*	475 (44%)	95 (63%)	231 (67%)
CSE†	444 (21%)	137 (12%)	10 (6%)	27 (8%)
ASD†	592 (29%)	247 (23%)	26 (17%)	49(14%)
ASA†	616 (30%)	352 (32%)	35 (23%)	67 (19%)
Avg. IR size	259 nt	171 nt	189 nt	188 nt
% id. with a human Ortholog^a^	--------	84%	85%	88%

IR (intron retained); CSE (Cassette exon); ASD (alternative splice donor); ASA (alternative splice acceptor).

† Values based on the number of genes.

* The percentage does not add up to 100 since one gene can have more than one event simultaneously.

a Values based on protein sequence.

In a previous work, we studied duplicated pseudogenic exons (*i.e.,* ΨEs, exons disabled by frameshifts and premature termination codons) on the same four genomes of our present study [13]. In order to evaluate the contribution of exon duplication and pseudogenization to the production of AS-NMD candidates, we compared the list of genes bearing a ΨE and the list of AS-NMD candidates. We found that only 7 genes are common to both lists in human, and 3 genes in mouse. Thus the vast majority of AS-NMD candidates are *not* made through exon duplication, followed by disablement. We also cross-referenced our gene list with the Ensembl pseudogenes annotation (Build 52) to verify whether any of our genes was annotated exclusively as a pseudogene. No pseudogene was found in our list, indicating that they are all alternatively-spliced forms of protein-coding genes. We additionally compared our list of AS-NMD candidates with the general annotation of the human genome in the Ensembl database (www.ensembl.org). We found that a small fraction (321 genes, ∼15%) in our list were also annotated as NMD-targeted transcripts by Ensembl.

Also, we wished to investigate whether AS-NMD events are associated with lower or higher gene expression levels. To do this, we used the number of ESTs/cDNAs as a broad indicator of expression level. In general, AS-NMD is associated with lower numbers of mapped ESTs/cDNAs (Supplementary Table S1A). For example, in human there are ∼75 transcripts mapped per gene, compared with ∼7 per gene for genes with AS-NMD events. Also, the substantial majority (>88%) of AS-NMD candidates have low numbers of ESTs/cDNAs (≤3 mapped sequences, Supplementary Table 1B).

### Conservation of NMD candidates and the genes that harbour them

We extracted orthology information about genes from the Ensembl database. Each AS-NMD candidate found in one of the three other species (mouse, rat and cow) was used as a query to find the corresponding ortholog in the human genome. Only matches annotated as ‘one2one’ (*i.e.*, the transcript is bi-directionally the best match between the two genomes) were counted as true orthologs.

We examined the human conservation of genes that harbour AS-NMD candidates in the three other genomes (mouse, rat and cow) (Tables 1–2). A large proportion of these genes have clear single orthologs in the human genome (87–91%). Of these orthologous human genes, 27–35% also harbour orthologous AS-NMD candidates. (See the Supplementary Table S2 for the full list of the AS-NMD candidate orthologs in each species, and Supplementary Table S3 for a complete breakdown of the different types of AS-NMD and their conservation patterns.) This degree of conservation is highly significant according to hypergeometric probability (P-values <10^−10^, treating the conserved cases as a sample of all human genes that have AS-NMD candidature).

Additional calculations for the number of conserved cases that would arise from random placement of the NMD features in human introns, also indicate that these results are highly significant. For example, for the smallest sample (human intron retentions whose NMD candidature is conserved in rat, 92 cases), we generated 1,000 random samples of 92 transcripts, with appropriate weighting for the number of introns in each transcript. These weightings were calculated simply through random sampling from the total list of introns in all human genes. Through this sampling procedure, given the frequency of NMD events in human, we found that only 1.3(±2.3) AS-NMD candidates would have conserved AS-NMD candidature by chance between human and rat (32 conserved cases are observed, Table 2).

This significant conservation of AS-NMD candidature arises both for the whole data set of AS-NMD candidates (Table 1), and for the subset that comprise intron retentions (IRs) only (Table 2). In addition, AS-NMD candidate genes without AS-NMD intron retentions, also exhibit highly significant conservation (not tabulated explicitly in Tables 1–2; P<<0.000001 for rat, mouse and cow using hypergeometric probability, as described above). Interestingly, however, only ∼13–22% of the IRs are conserved in the same position in the gene, indicating that there may just be selection pressure to maintain the NMD status, and not a specific retained intron.

Two models for AS-NMD candidates have recently been discussed, the ‘spurious transcript’ model, and the ‘regulatory model’ [12]. In the ‘spurious transcript’ model, the NMD function is to degrade costly-to-make and potentially toxic unwanted transcripts. Another prediction of this model is that AS-NMD should arise for rare transcripts, and be more common in recently-evolved exons; hence, it should not be conserved in other species. The alternative model is the ‘regulatory model’. In this model, the function of the NMD is to modulate gene expression. Although this model does not preclude multiple AS forms, it does not predict them. The regulatory model predicts that AS-NMD candidature should be conserved among distant species [12].

Our findings on AS-NMD candidate conservation are supportive of the regulatory model of NMD for a large subset of genes. Several previously well-documented examples have leant support to this theory. For instance, the NMD control of splicing in the splicing regulator (SR) genes, one of the most extensive studied cases of NMD controlling gene expression, is conserved across mammals and plants [14].

Our results seem to disagree with a previous study that found only ∼8% AS-NMD candidates in a pair of orthologs between human and mouse [12]. However, a closer look shows that our data is comparable. The authors in this previous study used only RefSeq mRNAs in their analysis. When we considered only RefSeq mRNAs, in our analysis, the number of pairs of orthologous AS-NMD candidates between human and mouse was 7%, in agreement with the previous study [12].

### Intron Retention (IR) Analysis

Intron retention is one of the least studied AS forms. One of the major reasons for this is the difficulty of differentiating real IRs from artifacts derived from unspliced or partially spliced pre-mRNA [15], [16]. However, previously it has been shown that retained introns seem to present sequence characteristics that distinguish them from non-retained introns, and also from protein-coding exons. These features include their average size, GC content and codon usage. In some cases the retained intron can also encode a protein domain or part of a domain [16]–[18].

**Figure 1:**
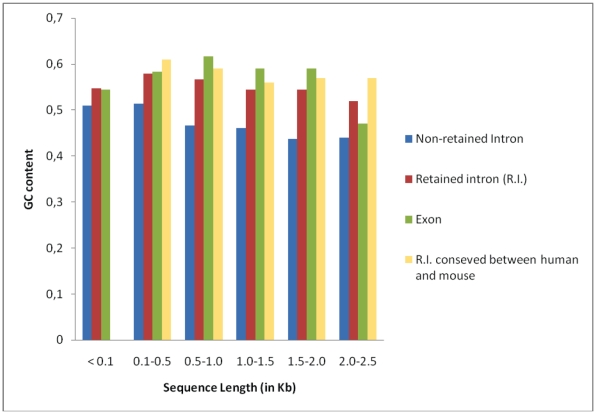
Mean GC content of retained introns, exons bordering retained introns and non-retained introns in human AS-NMD candidates. The data sets was divided into five categories to avoid bias due to their length. The data set of ‘Non-retained introns’ was generated by randomly picking a non-retained intron from the set of genes that contain retained introns, so that the same number of sequences is in each bin. In addition, we included bars in the plot for 'Retained introns that are conserved between human and mouse.

Here, we have assessed the AS-NMD retained introns in our data set for any evidence of selection pressures. One such source of evidence is deviation in GC content because of selection for transcriptional efficiency [15], [16], [19]. We analyzed deviation in GC content in the retained introns, as a function of intron length (Figure 1), to avoid GC content bias due to the size of the sequences. Firstly, we examined the following three data sets: retained introns, exons bordering retained introns, and non-retained introns. We divided the three data sets into categories according to their sequence length (Figure 1). Non-retained introns showed a statistically significant lower GC content in all classes of size compared with retained introns and exons (χ^2^ = 18.9, p<0.0001; χ^2^ = 25.6, p<0.0001, respectively). In contrast, retained introns showed a similar GC content compared with exons (χ^2^ = 0.67, p = 0.41). For comparison, we also included retained introns in genes that are orthologous AS-NMD candidates between human and mouse (Figure 1). Although, there is no retained intron shorter than 100 kb, all retained introns classes behaved more similarly to exons (χ^2^ = 0.21, p = 0.9) than to non-retained introns (χ^2^ = 34, p<0.0001).

To evaluate the codon usage of the retained introns we used a test described by Galante, *et al.* (2004, [15]) (for more details see the *Methods* section). We created 100 random sets of exons bordering retained introns and non-retained introns, each set containing 16970 codons (the number of codons in the retained intron set). We then put each sequence in frame and performed a pairwise comparison of the total codon table (61 amino acids) of the three data sets (Table 4). We find that retained introns and exons share more similarities with each other than with non-retained introns (Table 4). This indicates that the retained introns may have had phases of protein-coding ability before acquiring premature stop codons, or may still be protein-coding. The fact that codon usage of AS-NMD intron retentions is similar to the usage in exons indicates that it is difficult to de-couple AS-NMD-based regulation from the evolutionary dynamics of protein-coding intron retentions.

**Table 4:** Codon usage comparison of retained introns, exons and non-retained introns in human AS-NMD candidates.

	Retained introns and Non-retained introns	Retained introns and Exons	Non-retained introns and Exons
Codon table (Average χ^2^)*	1324±282	950±86	2531±310

*Comparison of the entire codon usage table (61 codons, 60 degrees of freedom) among three datasets.

We further analyzed the occurrence of stop codons in the retained introns (RIs), to analyse whether they may be able to become protein-coding through changing readong frames. On average, 95% of the RIs have a PTC in more than one frame and 90% of the RIs have a PTC in all three frames. Therefore, they are generally unlikely to be present in a functional, non-truncated protein.

We also assessed the ability of the AS-NMD candidate IRs to encode protein domains. To do this, we extracted three set of sequences: *intron retentions*, *exons* and sequences comprising retained introns and their 5′ and 3′ exons (‘*E-IR-E*’ sequences). These three sequence sets were compared against the Pfam database (as described in *Methods*). In total, we found 27 retained introns (in 27 genes) coding for a protein domain (17% of a total of 155 genes). Ten out of 27 retained introns encoded part of a domain also encoded by one of the flanking exons. Retained introns encoded for a different domain compared with their flanking exons in 14 cases and in three cases, *only* the retained intron encoded a Pfam domain. There was no case where the whole *E-IR-E* sequence encoded a domain.

The majority of the AS-NMD candidate ortholog pairs between human and mouse (153 out of 262) presented an IR in both species. As discussed above, the large number of AS-NMD candidates that are orthologously conserved between human and mouse is consistent with a widespread conserved mechanism to control gene expression through NMD.

We also annotated retained introns with PTCs that do not target the mRNA for NMD (termed ‘non-NMD’ retained introns) (Table 5). The number of genes bearing a non-NMD retained intron was between 2 and 4-fold higher than the number of genes with an AS-NMD candidate (the difference is statistically significant for all four genomes by χ^2^ test with P≤10^−7^). This indicates an apparent selection against PTC in NMD areas, and was also observed by us in a previous work [13], and by other authors as well [15]. The average size of non-NMD intron retentions is greater than AS-NMD candidates, although only in humans, this difference is statistically significant (χ^2^ = 19, p<0.0001) (Table 5).

**Table 5:** Retained introns (IRs) with a premature stop codon that not target the mRNA for NMD.

	*Homo sapiens*	*Mus musculus*	*Rattus norvegicus*	*Bos taurus*
Retained introns	5029	970	261	522
Number of genes with a IR.	3111	770	205	416
Avg. size of IR.	421 nt	180 nt	210 nt	202 nt
IR (3*n*)†	33.5%	34%	36%	35%

† Percentage of retained introns whose length is multiple of 3.

### Functional Annotation

We wished to determine the functional linkages of genes with AS-NMD as a subpopulation of all genes in an organism. Using the webtool FatiGO [20], we annotated the most common Gene Ontology terms [21], Biocarta pathways (http://www.biocarta.com/genes/index.asp), TRANSFAC [22] transcription factors, and KEGG [23] terms in each genome, and calculated which terms are overrepresented in genes with AS-NMD candidates. Significant over-representation was calculated using a Fisher's exact test with P' value threshold of <0.05, and a correction to P' for multiple hypothesis testing [20].

The term ‘*Nucleotide binding’* (GO:0000166) seems to be ubiquitous for AS-NMD candidates in the four genomes. Other abundant terms include those related to DNA interaction, DNA damage, cell cycle control and apoptosis (Table 6). Transferase and hydrolase activities were also common. The pattern of over- and under-representation of GO terms was similar for all four species. (The GO terms were simultaneously statistically significant in the four genomes only for non-corrected P values; see Table 6 for details.) This result shows that NMD may target different genes in different species, but these genes often belong to the same functional categories.

**Table 6:** Most represented GO terms per genome.

*Homo sapiens*	*Mus musculus*	*Rattus norvegicus*	*Bos taurus*
GO Molecular Function Level 3			
Hydrolase activity (GO:0016787) Nucleotide binding (GO:0000166) Lyase activity (GO:0016829) Oxidoreductase activity (GO:0016491) GTPase regulator activity (GO:0030695)	Nucleotide binding (GO:0000166) Hydrolase activity (GO:0016787) Transferase activity (GO:0016740) Helicase activity (GO:0004386) †Oxidoreductase activity (GO:0016491)†	Transferase activity (GO:0016740) † Amine transporter activity (GO:0005275) † Organic acid transporter activity (GO:0005342) † Nucleotide binding (GO:0000166) † Enzyme activator activity (GO:0008047) †	Structural constituent of ribosome (GO:0003735) † Nucleotide binding (GO:0000166) † Selenium binding (GO:0008430) † Transferase activity (GO:0016740) † Helicase activity (GO:0004386) †
GO Molecular Function Level 4			
Transferase activity, transferring phosphorus-containing groups (GO:0016772) Purine nucleotide binding (GO:0017076) Translation factor activity, nucleic acid binding (GO:0008135) Carbon-carbon lyase activity (GO:0016830) RNA binding (GO:0003723) †	Purine nucleotide binding (GO:0017076) Transferase activity, transferring phosphorus-containing groups (GO:0016772) Hydrolase activity, acting on acid anhydrides (GO:0016817) Ligase activity, forming carbon-oxygen bonds (GO:0016875) RNA binding (GO:0003723) †	Hydrolase activity, acting on glycosyl bonds (GO:0016798) † Transferase activity, transferring phosphorus-containing groups (GO:0016772) † Purine nucleotide binding (GO:0017076) † Carboxylic acid transporter activity (GO:0046943)† GTPase activator activity (GO:0005096) †	Purine nucleotide binding (GO:0017076)† Hydrolase activity, acting on glycosyl bonds (GO:0016798) † Calmodulin binding (GO:0005516) † Enzyme binding (GO:0019899) † Oxidoreductase activity, acting on peroxide as acceptor (GO:0016684) † ATPase inhibitor activity (GO:0042030)†

† Statistically significant (but for P value non-corrected for multiple hypotheses only).

We also performed a census of the most common protein domains in the genes with AS-NMD candidates (with all domains counted only *once* per gene). The most common Pfam protein domain was the WD-40 domain (except in rat) (Supplementary table S4). This domain is known to be involved in several cellular functions, such as signal transduction, cell-cycle control and apoptosis [24]. (Supplementary table S4).

In cow and rat, cancer-related pathways are significantly enriched (for P-values corrected for multiple hypothesis testing) according to KEGG classification. In mouse, besides cancer, AS-NMD candidates related with apoptosis are the most abundant. Human AS-NMD candidates are enriched for ‘glycine, serine and threonine metabolism’, ‘epithelial cell signaling in Helicobacter pylori infection’ and ‘androgen and estrogen metabolism’ KEGG pathways (Supplementary table S5). One role of NMD seems to be to protect individuals that are heterozygous for a PTC-containing allele; such an NMD-based mechanism has been demonstrated for genes associated with anemia, breast cancer, and diabetes type II [8], [25].

In the BioCarta pathway classification, we found five pathways in humans, enriched for AS-NMD candidates: antigen processing and presentation, inhibition of cellular proliferation by Gleevec, cell-to-cell adhesion signaling, integrin signaling pathway and role of Ran in mitotic spindle regulation (supplementary table S6).

Supplementary Figure S1 shows an example of a Biocarta pathway enriched for AS-NMD candidates. The drug Gleevec (also known as imatinib mesylate or STI-571) is considered one of the major breakthroughs in the treatment of cancer, especially CML, chronic myeloid leukemia. Gleevec acts on a molecular target by a mechanism that is more specific to cancer cells than traditional cytotoxic treatments [26]. We found four key genes in the ‘Inhibition of cellular proliferation by Gleevec’ pathway that are targets for NMD: breakpoint cluster region (BRC), mitogen-activated protein kinase 1 (MAP3K1), v-akt murine thymoma viral oncogene homolog 1 (Akt-1), and c-fos FBJ murine osteosarcoma viral oncogene homolog (FOS). The loss of expression of these genes is linked with several diseases and in some cases can be lethal. For example, it was found that loss of Akt1 resulted in defective ischemia and Vegf-induced angiogenesis and severe peripheral vascular disease in mice [27]. Loss of Mekk1 expression resulted in a greater apoptotic response of cells to hyperosmolarity and microtubule disruption [28].

We also calculated enrichments of transcription factor binding sites in the promoters of genes that target AS-NMD candidates (Supplementary Table S7). In human, the most significantly-enriched transcription factor binding site was for E2F1. Merdzhanova *et. al.* (2008, [29]), studied the association of E2F1 and the splicing regulator SC35 (a gene with an AS-NMD candidate). According to these authors, E2F1 upregulates the expression of SC35 in response to DNA-damaging agents; they also show that SC35 is required for apoptosis in response to these agents. The apoptotic mechanism involves the pro-apoptotic alternative splice form of the gene Bcl-x (also a AS-NMD candidate) that is in turn controlled by SC35 [29]. Expression of the NMD-targeted form of SC35 could switch on the overexpression of anti-apoptotic splice variants of genes like Bcl-x and caspase. Such alteration is believed to contribute to the resistance of tumor cells to chemotherapy [30], [31].

We compared the representation of AS-NMD candidate genes in the OMIM database with the overall representation of the human genome in the same database. The difference of representation of the two sets in the OMIM database is highly statistically significant (χ^2^ = 129, p<0.0001). While 35% of the human gene complement is present in OMIM, the proportion of human AS-NMD candidate genes in OMIM is 50%. NMD-associated diseases often result from insufficient levels of full-length protein. Therefore, these diseases are generally due to two defects in gene expression: degradation of the newly synthesized PTC-containing mRNA by NMD, and failure of the abnormally low level of PTC-containing mRNA that escaped NMD to generate full-length, and thus, functional protein.

In summary, these analyses indicate that similar functional categories and protein domains are enriched in genes with AS-NMD in all four genomes. Also, in general, KEGG pathways related to cancer are enriched, and AS-NMD genes are more likely to be associated with diseases in the OMIM database. We discussed a specific example of a pathway with several AS-NMD candidates, that is important for cancer treatment, that we found in the Biocarta classification.

### Mapping AS-NMD candidates to the MS PRIDE database

Since 5 to 25% of PTC-containing mRNAs fail to elicit NMD and are consequently translated into truncated proteins [32], several therapies have been developed specially to suppress in-frame PTC [33], [34]. Some criticism of nonsense-suppressor therapies is that new diseases can be created even if only a single C-terminally extended protein from one of the many cellular transcripts were to accumulate to a toxic level. Furthermore, another problem is the unknown consequences of failure to eliminate naturally-occurring NMD targets [35].

To quantify the frequency with which the expressed AS-NMD candidates escape NMD and are translated into proteins we mapped each human translated AS-NMD candidate to ∼550,000 peptides extracted from the repository of mass spectrometry-derived proteomics data (PRIDE) [36].

After filtering out the matches for uniqueness and size (peptides less than 7 amino acids long and peptides matching another gene were discarded) we found 205 AS events in 194 genes with peptide matches in the alternatively-spliced area (hereafter referred as AS-NMD-PRIDE genes) (Table 7). This is ∼10% of the total number of genes targeted to NMD (Tables 1–2).

**Table 7:** Comparison between human AS-NMD candidates with and without matches in PRIDE database.

*Homo sapiens*	AS events	ESTs/cDNAs/mRNAs supporting the AS	Number Genes	Orthologs in another genome	AS-NMD candidates with an orthologous AS-NMD candidate in another genome	AS-NMD candidates with a IR
AS-NMD candidate with match in PRIDE	205	385	194	159 (mouse) 139 (rat) 156 (cow)	30 (mouse) 5 (rat) 13 (cow)	110
AS-NMD candidate without match	2331	13958	1857	1560 (mouse) 1425 (rat) 1478 (cow)	232 (mouse) 40 (rat) 92 (cow)	884

The majority (71%–81%) of the human AS-NMD-PRIDE genes possess an ortholog in one of the other species studied. We also compared the ‘AS-NMD-PRIDE’ genes with the remaining AS-NMD candidates (AS-NMD candidates with no match in PRIDE database). Surprisingly, the proportion with conserved AS-NMD orthologs was nearly the same for both sets of genes. For example, in the AS-NMD-PRIDE set 18%, 3% and 8% of the human orthologs in mouse, rat and cow respectively were also targeted for NMD. For the remaining AS-NMD candidates, the proportion was 14%, 3% and 6% (in mouse, rat and cow). More than half of the AS-NMD-PRIDEs come from AS forms containing retained introns (56%), while in AS-NMD candidates the frequency is 47%. Interestingly, the retained introns with PRIDE matches do not show any significant deviation with respect to GC% (Supplementary Table S8).

The AS-NMD-PRIDE set of genes were significantly enriched for ‘*protein metabolic process*’ (GO:0019538), ‘*cellular macromolecule metabolic process*’ (GO:0044260) GO biological process and ‘*structural constituent of ribosome*’ (GO:0003735) GO molecular function, compared with the whole human genome. The last GO term was also enriched when AS-NMD-PRIDE set of genes was compared with the general AS-NMD candidates set.

**Figure 2:**
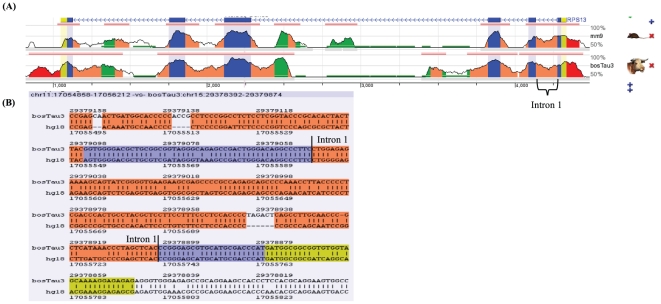
The RPS3 gene. (A) Conservation of the gene RPS13 among human, mouse and cow. Yellow bars, UTRs; Blue bars exons; Orange bars, Intron; Green bar, repetitive elements. The conserved retained intron is indicated (braces). (B) Alignment of UTR-Exon1-Intron1-Exon2-Intron2. The start and end of the conserved intron is indicated by vertical bars.

**Figure 3:**
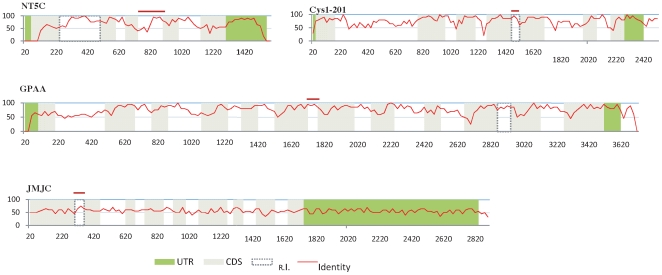
Percentage of identity in a window of 20 nucleotides of five pairs of AS-NMD candidates orthologs between human and mouse. The red bar above each graph represents the retained intron in the mouse AS-NMD candidate (IR  =  retained intron in the human genome).

Among the genes present in the ‘*structural constituent of ribosome*’ GO term we found two cases in which the ortholog gene was also targeted for NMD: the gene RPL5 in mouse and the bovine RPS13. The human and cow RPS13 presented a retention of intron 1 that targets its mRNA for NMD. Using the ECR browser [37] to generate an alignment of the RPS13, we could observe a high GC content in the intron 1 (>65%), as well as small size (141 nt), and a high conservation among human, mouse and cow (Figure 2). Taken together, these are the typical symptoms of a non-spurious retained intron. Recent study showed that the presence of intron 1 in the human RPS13 reduced its expression by a factor of four [38]. Apparently, the gene RPS13 can regulate its protein level via a feedback mechanism. The ribosomal protein S3 was found to inhibit the excision of the intron 1 from the rpS13 pre-mRNA. This protein binds specifically the intron fragment near the 5′ and 3′ splice site, therefore conferring protection against cleavage by ribonucleases [38]. Although the extraribosomal function of rpS13 is still unclear, a study suggested that this gene (along with rpL23) promotes multidrug resistance in gastric cancer cells by suppressing apoptosis [39]. A similar pattern of proteins regulating their own splicing and generating aberrant mRNA was also found in *Saccharomyces cerevisiae* [40] and in *C. elegans* [41] which suggests that NMD plays a important role in gene regulation. In Figure 3, we have illustrated five other examples of retained introns, that are conserved in mouse; note that each of these cases is in a gene structure with a large number of introns.

### Conclusions

In this work, we have annotated and analyzed alternative splicing coupled with nonsense-mediated decay in four mammalian species: human, mouse, rat and cow. We have shown that alternative splicing coupled with NMD occurs in 2 to 10% of the known annotated genes in each genome, and that there is a highly significant conservation of AS-NMD candidature in genes across the four mammals. Human AS-NMD candidates seem to have a large number of orthologs in mouse (∼82%), and at least 15% of those orthologs are also AS-NMD candidates in another species. We think that, in the future, increased sampling of transcripts will lead to increased discovery of conservation of specific AS-NMD events between different organisms. It is possible that there are many such AS-NMD features in genes, but that we have not detected all of them because of insufficient sampling. Also, the AS-NMD events may be designed to be easily turned ‘on’ or ‘off’ (*i.e.*, the ability to include the AS-NMD feature may be easily modified over evolutionary time).

Although we only analyzed AS-NMD candidates with in-frame PTCs in this study, we have demonstrated that intron retention plays an important role in producing AS-NMD candidates in all four species. A large fraction (58 to 81%, depending on the species) of conserved AS-NMD candidates are intron retentions. The retained introns also possess features that distinguish them from non-retained introns, such as smaller size, higher GC content and codon usage similar to exons. The latter indicates that retained introns tend to have phases of protein-coding ability before acquiring stop codons; it is thus difficult to de-couple this phenomenon from the additional role of such transcripts as NMD candidates.

The AS-NMD candidate genes also showed a similar pattern of Gene Ontology enrichment in all four species. Genes linked to nucleotide interaction and cell fate (apoptosis) were the most abundant to produce AS-NMD candidates. KEGG and Biocarta pathways also showed an over-representation of pathways linked with cancer and genetic disorders for such AS-NMD-making genes. Thus, NMD appears to have a significant role in controlling the expression of aberrant proteins in these pathways, and prevents their deleterious effects.

We gathered evidence of translation of at least 10% of all AS-NMD candidates in the human genome. Such cases, are not, however, especially conserved in comparison to other AS-NMD candidates. We cannot determine here whether such truncated proteins have a function in human cells; they may just be ‘junk’ polypeptides that are degraded at a later stage. However, because retained introns with premature stop codons have similar codon usage to exons, this strongly indicates that some NMD substrates have at least been protein-coding in the recent evolutionary past.

Furthermore, in one of the AS-NMD candidates, the gene RPS13, we found evidences of a conserved mechanism of autoregulation, between human and cow, in which the protein inhibits the excision of one of its introns, creating a splice form that is targeted for NMD.

Alternative splicing coupled with NMD seems to act on a wide range of genes, but, however, only in a few significantly over-represented functions across mammalian genomes. The data herein provide statistical evidence for conserved regulation *via* AS-NMD candidature across *Mammalia*, for a large subset of genes.

## Methods

### Genome data

The genome sequences and annotations of four mammals (human, cow, mouse and rat) were downloaded from the Ensembl Web site (http://www.ensembl.org), in March 2009. The genome assemblies are: human = Homo_sapiens.NCBI36.52; cow = Bos_taurus.Btau_4.0.52; mouse = Mus_musculus.NCBIM37.52 and rat = Rattus_norvegicus.RGSC3.4.52. These genomes were chosen based on two criteria: *(i)* high coverage (>7X), and *(ii)* large number of cDNAs and ESTs sequences available (covering >85% of annotated genes).

The cDNA/EST/mRNA data were downloaded from the Refseq database (ftp://ftp.ncbi.nih.gov/refseq), the H-invitational database (http://h-invitational.jp/hinv/ahg-db), the Unigene compendium (ftp://ftp.ncbi.nih.gov/repository/Unigene; Build #217) and the dbEST data set (ftp://ftp.ncbi.nih.gov/repository/dbEST/), in March 2009. At the time of downloading, the dbEST database contained deposited data from next-generation sequencing in addition to EST sequencing data from older techniques.

### Identification of alternative splice forms with an in-frame premature stop codon targeting the mRNA for nonsense-mediated decay

#### (I) Reference identification

We identified the reference mRNA (an mRNA that aligns with 100% coverage and identity to an annotated protein) of each human, mouse, rat and cow transcripts. A bl2seq alignment [42] was performed between mRNAs from Refseq, Unigene and H-invitational (human genome only) and each protein in the Ensembl database. Transcripts without a reference mRNA were discarded from our analysis.

The genomic loci were identified and extracted from the Ensembl annotations and expanded by 500 nucleotides 5′ and 3′ of the locus, to allow for variation in the transcriptional start and polyadenylation sites [25]. Each reference mRNA was aligned with its genomic locus using GMAP [43].

#### (II) Mapping

To identify AS forms we aligned each genomic locus that has a reference mRNA, against all mRNAs and ESTs/cDNAs from the Refseq, Unigene, dbEST and H-invitational databases (this last database is for human only). Each alignment was parsed into a set of splice junction coordinates. Alignments where the coverage was <0.96 of the length of the mRNA or EST and <98% sequence identity were discarded. We also discarded ESTs that aligned to the opposite strand. In cases were a mRNA/cDNA/EST mapped to more than one gene, the best mapping was considered the correct one. If a mapping presented the same percentage of identity and coverage to more than one gene, the mapping was discarded.

#### (III) Coding sequence (CDS) annotation

We performed a new round of alignments to identify the coding region (CDS) of each mapped transcript. Using the program Exonerate [44], we aligned each transcript retrieved from the previous mapping, against the encoded protein extracted from the Ensembl annotation.

#### (IV) Nonsense-mediated decay targeting annotation

We compared each mapped splice junction with the reference mRNA splice junctions. If a splice form mapped >10 nt from the reference junction, it was annotated as an alternative splice form. We verify the presence of PTCs targeting the mRNA/EST for NMD, using the 55-nucleotide rule [6]. Using the CDS annotation, we checked the mRNA/EST sequence for an in-frame stop codon 55 nucleotides or more 5′ to an exon-exon junction.

### Intron retention (IR) analysis

To verify whether genes targeted for NMD due to IR were caused by an unspliced or partially-spliced pre-mRNA, we analyzed several features of our sequences and compared them with their flanking exons, and with non-retained introns. Some of our analysis followed the analysis described by Galante, *et al.* (2004) [15].

#### Codon Usage/Bias

We assess the codon usage and codon bias of retained introns, exons bordering retained introns and non-retained introns. We only used AS-NMD candidates with at least one full length cDNA containing the IR. The frame of non-retained introns and retained introns was defined by the frame of the exon 5′ to it.

We analyzed the general codon bias of all 61 codons in the three sets. We performed 100 pairwise comparisons of the distribution of 16,970 codons (number of codons in the retained intron set), along with 61 possible codons to calculate the average χ^2^ and standard deviations thereof.

#### Pfam domain analysis

DNA sequences from retained introns, exons bordering retained introns and *exon–intron retention–exon* (*‘E-IR-E’*) sequences were compared to the Pfam database using BLASTx program (e-value 10^−2^). Only genes with a full-length cDNA/EST and with a retained intron entirely in the CDS were used in this analysis (155 in total).

#### GC content

To assess the GC content of the retained introns, exons bordering retained introns and non-retained introns, we created three datasets with the same number of sequences divided into five classes according to their length. The non-retained introns were randomly picked from the set of genes containing the retained introns in order to avoid any kind of bias toward a specific type of gene. A χ^2^ test was used to evaluate the differences between each dataset.

### Orthologs

The information about pairs of orthologs between human and the other species was extracted from the Biomart query system in the Ensembl database. We only considered two genes as being orthologs when they mapped in a one-to-one way.

### Functional Annotation

Gene Ontology (GO [21]) functional categories, transcription factors TRANSFAC [22] and KEGG [23] and BioCarta (http://www.biocarta.com/genes/index.asp) terms, were retrieved from the FatiGo database [20]. We performed a functional enrichment analysis of our list of AS-NMD candidates against the whole genome annotation of each species. Significant overrepresentation was calculated using a Fisher's exact test with P' <0.05, and a correction to P' for multiple hypothesis testing [20]. Human AS-NMD candidates were cross-referenced with the NCBI OMIM database (http://www.ncbi.nlm.nih.gov/sites/entrez?db=omim), to identify linkage to genetic disorders.

### Mapping peptides from mass spectrometry-derived proteomics data to AS_NMD candidates

We mapped all human AS-NMD candidates to >600,000 peptides from mass spectrometry, extracted from the PRIDE database [36]. These peptides are derived from a wide variety of human samples, as follows: blood plasma, peripheral blood mononuclear cells, erythrocytes, platelets, brain, HeLa cells, K-562 cells, HEK-293 cells, melanocytes, placenta, breast cancer cells, bone marrow cancer cells, JURKAT cells, liver, cerebrospinal fluid, heart and saliva. Each transcript was translated in the 3 frames and checked against the PRIDE peptides. We derived a Perl program to check the occurrence of peptides upstream of the PTC. We filtered out any matching peptide smaller than 7 amino acids, and also any peptides matching to another human gene or protein.

## Supporting Information

Figure S1 Gleevec pathway with genes with NMD candidate transcripts labelled.
(0.63 MB EPS)

Table S1 Summary of numbers of ESTs/cDNAs mapping to genes.
(0.04 MB DOC)

Table S2 Orthologs of human NMD candidates.
(0.47 MB DOC)

Table S3 Summary of the different types of NMD candidate.
(0.03 MB DOC)

Table S4 Top five domain types in NMD candidates in the four mammals studied.
(0.05 MB DOC)

Table S5 Overrepresented KEGG pathways
(0.04 MB DOC)

Table S6 Overrepresented Biocarta pathways
(0.03 MB DOC)

Table S7 Overrepresented transcription factors
(0.07 MB DOC)

Table S8 Classification of introns by size
(0.03 MB DOC)

## References

[1] Zavolan M, van Nimwegen E, Gaasterland T (2002). Splice variation in mouse full-length cDNAs identified by mapping to the mouse genome.. Genome Res.

[2] Brett D, Pospisil H, Valcarcel J, Reich J, Bork P (2002). Alternative splicing and genome complexity.. Nat Genet.

[3] Johnson JM, Castle J, Garrett-Engele P, Kan Z, Loerch PM (2003). Genome-wide survey of human alternative pre-mRNA splicing with exon junction microarrays.. Science.

[4] Tazi J, Bakkour N, Stamm S (2009). Alternative splicing and disease.. Biochim Biophys Acta.

[5] Lewis BP, Green RE, Brenner SE (2003). Evidence for the widespread coupling of alternative splicing and nonsense-mediated mRNA decay in humans.. Proc Natl Acad Sci U S A.

[6] Chang YF, Imam JS, Wilkinson MF (2007). The nonsense-mediated decay RNA surveillance pathway.. Annu Rev Biochem.

[7] Silva AL, Romao L (2009). The mammalian nonsense-mediated mRNA decay pathway: to decay or not to decay! Which players make the decision?. FEBS Lett.

[8] Holbrook JA, Neu-Yilik G, Hentze MW, Kulozik AE (2004). Nonsense-mediated decay approaches the clinic.. Nat Genet.

[9] Le Hir H, Izaurralde E, Maquat LE, Moore MJ (2000). The spliceosome deposits multiple proteins 20-24 nucleotides upstream of mRNA exon-exon junctions.. Embo J.

[10] Saltzman AL, Kim YK, Pan Q, Fagnani MM, Maquat LE (2008). Regulation of multiple core spliceosomal proteins by alternative splicing-coupled nonsense-mediated mRNA decay.. Mol Cell Biol.

[11] Kaygun H, Marzluff WF (2005). Regulated degradation of replication-dependent histone mRNAs requires both ATR and Upf1.. Nat Struct Mol Biol.

[12] Zhang Z, Xin D, Wang P, Zhou L, Hu L (2009). Noisy splicing, more than expression regulation, explains why some exons are subject to nonsense-mediated mRNA decay.. BMC Biol.

[13] Morais DD, Harrison PM (2009). Genomic evidence for non-random endemic populations of decaying exons from mammalian genes.. BMC Genomics.

[14] Lareau LF, Inada M, Green RE, Wengrod JC, Brenner SE (2007). Unproductive splicing of SR genes associated with highly conserved and ultraconserved DNA elements.. Nature.

[15] Galante PA, Sakabe NJ, Kirschbaum-Slager N, de Souza SJ (2004). Detection and evaluation of intron retention events in the human transcriptome.. Rna.

[16] Kurmangaliyev YZ, Gelfand MS (2008). Computational analysis of splicing errors and mutations in human transcripts.. BMC Genomics.

[17] Hiller M, Huse K, Platzer M, Backofen R (2005). Non-EST based prediction of exon skipping and intron retention events using Pfam information.. Nucleic Acids Res.

[18] Jaillon O, Bouhouche K, Gout JF, Aury JM, Noel B (2008). Translational control of intron splicing in eukaryotes.. Nature.

[19] Berget SM (1995). Exon recognition in vertebrate splicing.. J Biol Chem.

[20] Al-Shahrour F, Minguez P, Vaquerizas JM, Conde L, Dopazo J (2005). BABELOMICS: a suite of web tools for functional annotation and analysis of groups of genes in high-throughput experiments.. Nucleic Acids Res.

[21] Harris MA, Clark J, Ireland A, Lomax J, Ashburner M (2004). The Gene Ontology (GO) database and informatics resource.. Nucleic Acids Res.

[22] Wingender E (2008). The TRANSFAC project as an example of framework technology that supports the analysis of genomic regulation.. Brief Bioinform.

[23] Kanehisa M, Goto S, Furumichi M, Tanabe M, Hirakawa M (2009). KEGG for representation and analysis of molecular networks involving diseases and drugs.. Nucleic Acids Res.

[24] Neer EJ, Schmidt CJ, Nambudripad R, Smith TF (1994). The ancient regulatory-protein family of WD-repeat proteins.. Nature.

[25] Green RE, Lewis BP, Hillman RT, Blanchette M, Lareau LF (2003). Widespread predicted nonsense-mediated mRNA decay of alternatively-spliced transcripts of human normal and disease genes.. Bioinformatics.

[26] Kuenen BC, Pinedo HM (2003). [New oncological treatment principle with imatinib].. Ned Tijdschr Geneeskd.

[27] Peng XD, Xu PZ, Chen ML, Hahn-Windgassen A, Skeen J (2003). Dwarfism, impaired skin development, skeletal muscle atrophy, delayed bone development, and impeded adipogenesis in mice lacking Akt1 and Akt2.. Genes Dev.

[28] Yujiri T, Sather S, Fanger GR, Johnson GL (1998). Role of MEKK1 in cell survival and activation of JNK and ERK pathways defined by targeted gene disruption.. Science.

[29] Merdzhanova G, Edmond V, De Seranno S, Van den Broeck A, Corcos L (2008). E2F1 controls alternative splicing pattern of genes involved in apoptosis through upregulation of the splicing factor SC35.. Cell Death Differ.

[30] Mercatante D, Kole R (2000). Modification of alternative splicing pathways as a potential approach to chemotherapy.. Pharmacol Ther.

[31] Hayes GM, Carrigan PE, Beck AM, Miller LJ (2006). Targeting the RNA splicing machinery as a novel treatment strategy for pancreatic carcinoma.. Cancer Res.

[32] Stephenson LS, Maquat LE (1996). Cytoplasmic mRNA for human triosephosphate isomerase is immune to nonsense-mediated decay despite forming polysomes.. Biochimie.

[33] Kerem E (2004). Pharmacologic therapy for stop mutations: how much CFTR activity is enough?. Curr Opin Pulm Med.

[34] Ainsworth C (2005). Nonsense mutations: running the red light.. Nature.

[35] Kuzmiak HA, Maquat LE (2006). Applying nonsense-mediated mRNA decay research to the clinic: progress and challenges.. Trends Mol Med.

[36] Vizcaino JA, Cote R, Reisinger F, Barsnes H, Foster JM The Proteomics Identifications database: 2010 update.. Nucleic Acids Res.

[37] Ovcharenko I, Nobrega MA, Loots GG, Stubbs L (2004). ECR Browser: a tool for visualizing and accessing data from comparisons of multiple vertebrate genomes.. Nucleic Acids Res.

[38] Malygin AA, Parakhnevitch NM, Ivanov AV, Eperon IC, Karpova GG (2007). Human ribosomal protein S13 regulates expression of its own gene at the splicing step by a feedback mechanism.. Nucleic Acids Res.

[39] Shi Y, Zhai H, Wang X, Han Z, Liu C (2004). Ribosomal proteins S13 and L23 promote multidrug resistance in gastric cancer cells by suppressing drug-induced apoptosis.. Exp Cell Res.

[40] Fewell SW, Woolford JL (1999). Ribosomal protein S14 of Saccharomyces cerevisiae regulates its expression by binding to RPS14B pre-mRNA and to 18S rRNA.. Mol Cell Biol.

[41] Mitrovich QM, Anderson P (2000). Unproductively spliced ribosomal protein mRNAs are natural targets of mRNA surveillance in C. elegans.. Genes Dev.

[42] Tatusova TA, Madden TL (1999). BLAST 2 Sequences, a new tool for comparing protein and nucleotide sequences.. FEMS Microbiol Lett.

[43] Wu TD, Watanabe CK (2005). GMAP: a genomic mapping and alignment program for mRNA and EST sequences.. Bioinformatics.

[44] Slater GS, Birney E (2005). Automated generation of heuristics for biological sequence comparison.. BMC Bioinformatics.

