# Supplementary material for: Large-Scale Evidence for Conservation of NMD Candidature Across Mammals
Source: PLoS One. 2010 Jul 21;5(7):e11695. doi: 10.1371/journal.pone.0011695 (PMC2908137; doi:10.1371/journal.pone.0011695)
Supplement: Table S1 — Summary of numbers of ESTs/cDNAs mapping to genes. (0.04 MB DOC) [file pone.0011695.s002.doc]

| **Supplementary Table 1(A). Total number of mapped sequences** | | | | |
| --- | --- | --- | --- | --- |
|  | Genes with mappings | EST/mRNA/cDNAs mapped § | # of mapped transcripts per gene | # of mapped transcripts per AS-NMD gene |
| *Homo_sapiens* | 22121 | 1663325 | 75.2 | 7.0 |
| *Mus_musculus* | 29616 | 452495 | 15.3 | 1.5 |
| *Rattus_norvegicus* | 14442 | 47707 | 3.3 | 1.3 |
| *Bos_taurus* | 13597 | 113279 | 8.3 | 1.8 |

§ The total number of EST/cDNA transcripts per gene with mappings.

| **Supplementary Table 1(B). Total number of mapped sequences** ^§^ | | | |
| --- | --- | --- | --- |
|  | From 1–3 ESTs/cDNAs | From 4–10 ESTs/cDNAs | > 10 ESTs/cDNAs |
| *Homo sapiens* | 1804 (88%) | 205 (10%) | 42 (2%) |
| *Mus musculus* | 1005 (94%) | 61 (5.7%) | 3 (0.3%) |
| *Rattus norvegicus* | 147 (98.6%) | 2 (1.4%) | - |
| *Bos taurus* | 321 (93.9%) | 19 (5.5%) | 2 (0.6%) |

§ Percentages are of the total number of genes with AS-NMD according to table 1(A)(3)
