# Supplementary material for: Large-Scale Evidence for Conservation of NMD Candidature Across Mammals
Source: PLoS One. 2010 Jul 21;5(7):e11695. doi: 10.1371/journal.pone.0011695 (PMC2908137; doi:10.1371/journal.pone.0011695)
Supplement: Table S2 — Orthologs of human NMD candidates. (0.47 MB DOC) [file pone.0011695.s003.doc]

|  | Table. S2 Human orthologs | | | | |  | |
| --- | --- | --- | --- | --- | --- | --- | --- |
|  | ***Mus musculus*** | | | | |  | |
|  | Transcript | Gene Name | Chr | Human ortholog ID | Description |  | |
|  | ENSMUST00000000505 | Mcm7 | 5 | ENSG00000166508 | minichromosome maintenance deficient 7 (S. cerevisiae) Gene |  | |
|  | ENSMUST00000001331 | Myg1 | 15 | ENSG00000139637 | melanocyte proliferating gene 1 Gene |  | |
|  | ENSMUST00000001335 | Pfdn5 | 15 | ENSG00000123349 | prefoldin 5 Gene |  | |
|  | ENSMUST00000001454 | 0610031J06Rik | 3 | ENSG00000198715 | RIKEN cDNA 0610031J06 gene Gene |  | |
|  | ENSMUST00000001801 | Tcirg1 | 19 | ENSG00000110719 | T-cell, immune regulator 1, ATPase, H+ transporting, lysosomal V0 protein A3 Gene |  | |
|  | ENSMUST00000002079 | Plxnb3 | X | ENSG00000198753 | plexin B3 Gene |  | |
|  | ENSMUST00000002400 | 1810034K20Rik | 14 | ENSG00000213920 | RIKEN cDNA 1810034K20 gene Gene |  | |
|  | ENSMUST00000003183 | Ppp5c | 7 | ENSG00000011485 | protein phosphatase 5, catalytic subunit Gene |  | |
|  | ENSMUST00000003284 | Irf3 | 7 | ENSG00000126456 | interferon regulatory factor 3 Gene |  | |
|  | ENSMUST00000003550 | Ncstn | 1 | ENSG00000162736 | nicastrin Gene |  | |
|  | ENSMUST00000003857 | Shkbp1 | 7 | ENSG00000160410 | Sh3kbp1 binding protein 1 Gene |  | |
|  | ENSMUST00000004508 | Tmed4 | 11 | ENSG00000158604 | transmembrane emp24 protein transport domain containing 4 Gene |  | |
|  | ENSMUST00000004786 | Polr2e | 10 | ENSG00000099817 | polymerase (RNA) II (DNA directed) polypeptide E Gene |  | |
|  | ENSMUST00000005825 | Pan2 | 10 | ENSG00000135473 | PAN2 polyA specific ribonuclease subunit homolog (S. cerevisiae) Gene |  | |
|  | ENSMUST00000006061 | Pex1 | 5 | ENSG00000127980 | plasmacytoma expressed transcript 1 Gene |  | |
|  | ENSMUST00000006377 | Zbtb17 | 4 | ENSG00000116809 | zinc finger and BTB domain containing 17 Gene |  | |
|  | ENSMUST00000006444 | Tep1 | 14 | ENSG00000129566 | telomerase associated protein 1 Gene |  | |
|  | ENSMUST00000007042 | Ik | 18 | ENSG00000113141 | IK cytokine Gene |  | |
|  | ENSMUST00000007799 | Cav1 | 6 | ENSG00000105974 | caveolin 1, caveolae protein Gene |  | |
|  | ENSMUST00000009236 | Derl3 | 10 | ENSG00000099958 | Der1-like domain family, member 3 Gene |  | |
|  | ENSMUST00000010348 | Fdx1l | 9 | ENSG00000167807 | ferredoxin 1-like Gene |  | |
|  | ENSMUST00000010506 | Rdm1 | 11 | ENSG00000187456 | RAD52 motif 1 Gene |  | |
|  | ENSMUST00000011623 | Dennd1c | 17 | ENSG00000205744 | DENN/MADD domain containing 1C Gene |  | |
|  | ENSMUST00000012580 | Hps3 | 3 | ENSG00000163755 | Hermansky-Pudlak syndrome 3 homolog (human) Gene |  | |
|  | ENSMUST00000013299 | E130303B06Rik | 8 | ENSG00000124074 | RIKEN cDNA E130303B06 gene Gene |  | |
|  | ENSMUST00000013773 | Cad | 5 | ENSG00000084774 | congenital cataract Gene |  | |
|  | ENSMUST00000014927 | Plekhg4 | 8 | ENSG00000196155 | pleckstrin homology domain containing, family G (with RhoGef domain) member 4 Gene |  | |
|  | ENSMUST00000015017 | Surf2 | 2 | ENSG00000148291 | surfeit gene 2 Gene |  | |
|  | ENSMUST00000015291 | Lgals4 | 7 | ENSG00000171747 | lectin, galactose binding, soluble 4 Gene |  | |
|  | ENSMUST00000015435 | Gdi1 | X | ENSG00000203879 | guanosine diphosphate (GDP) dissociation inhibitor 1 Gene |  | |
|  | ENSMUST00000015540 | Cd83 | 13 | ENSG00000112149 | CD83 antigen Gene |  | |
|  | ENSMUST00000015791 | Lama5 | 2 | ENSG00000130702 | laminin, alpha 5 Gene |  | |
|  | ENSMUST00000017354 | Med24 | 11 | ENSG00000008838 | mediator complex subunit 24 Gene |  | |
|  | ENSMUST00000017904 | Ctsa | 2 | ENSG00000064601 | cathepsin A Gene |  | |
|  | ENSMUST00000018311 | Stard3 | 11 | ENSG00000131748 | START domain containing 3 Gene |  | |
|  | ENSMUST00000018593 | Rpain | 11 | ENSG00000129197 | RPA interacting protein Gene |  | |
|  | ENSMUST00000018851 | Dync1h1 | 12 | ENSG00000197102 | dynein cytoplasmic 1 heavy chain 1 Gene |  | |
|  | ENSMUST00000019169 | Use1 | 8 | ENSG00000053501 | unconventional SNARE in the ER 1 homolog (S. cerevisiae) Gene |  | |
|  | ENSMUST00000019382 | 1600014K23Rik | 8 | ENSG00000099797 | glycoprotein, synaptic 2 Gene |  | |
|  | ENSMUST00000019447 | Psmc3ip | 11 | ENSG00000131470 | proteasome (prosome, macropain) 26S subunit, ATPase 3, interacting protein Gene |  | |
|  | ENSMUST00000019464 | Noxo1 | 17 | ENSG00000196408 | NADPH oxidase organizer 1 Gene |  | |
|  | ENSMUST00000019882 | Polr2i | 7 | ENSG00000105258 | polymerase (RNA) II (DNA directed) polypeptide I Gene |  | |
|  | ENSMUST00000019962 | Cd164 | 10 | ENSG00000135535 | CD164 antigen Gene |  | |
|  | ENSMUST00000019965 | Smpd2 | 10 | ENSG00000135587 | sphingomyelin phosphodiesterase 2, neutral Gene |  | |
|  | ENSMUST00000019967 | Mical1 | 10 | ENSG00000135596 | microtubule associated monoxygenase, calponin and LIM domain containing 1 Gene |  | |
|  | ENSMUST00000020109 | Actr6 | 10 | ENSG00000075089 | ARP6 actin-related protein 6 homolog (yeast) Gene |  | |
|  | ENSMUST00000020340 | Pcsk4 | 10 | ENSG00000115257 | proprotein convertase subtilisin/kexin type 4 Gene |  | |
|  | ENSMUST00000020383 | Atp8b3 | 10 | ENSG00000130270 | ATPase, class I, type 8B, member 3 Gene |  | |
|  | ENSMUST00000020420 | Ap3d1 | 10 | ENSG00000065000 | adaptor-related protein complex 3, delta 1 subunit Gene |  | |
|  | ENSMUST00000020437 | Mdm1 | 10 | ENSG00000111554 | transformed mouse 3T3 cell double minute 1 Gene |  | |
|  | ENSMUST00000020640 | Gnb2l1 | 11 | ENSG00000204628 | guanine nucleotide binding protein (G protein), beta polypeptide 2 like 1 Gene |  | |
|  | ENSMUST00000020705 | Pes1 | 11 | ENSG00000100029 | pescadillo homolog 1, containing BRCT domain (zebrafish) Gene |  | |
|  | ENSMUST00000020767 | Polm | 11 | ENSG00000122678 | polymerase (DNA directed), mu Gene |  | |
|  | ENSMUST00000020770 | Mrps24 | 11 | ENSG00000062582 | mitochondrial ribosomal protein S24 Gene |  | |
|  | ENSMUST00000020846 | Srebf1 | 11 | ENSG00000072310 | sterol regulatory element binding transcription factor 1 Gene |  | |
|  | ENSMUST00000021062 | Ddx5 | 11 | ENSG00000108654 | DEAD (Asp-Glu-Ala-Asp) box polypeptide 5 Gene |  | |
|  | ENSMUST00000021082 | Nt5c | 11 | ENSG00000125458 | 5',3'-nucleotidase, cytosolic Gene |  | |
|  | ENSMUST00000021339 | 6530401N04Rik | 12 | ENSG00000129480 | RIKEN cDNA 6530401N04 gene Gene |  | |
|  | ENSMUST00000021666 | Abcd4 | 12 | ENSG00000119688 | ATP-binding cassette, sub-family D (ALD), member 4 Gene |  | |
|  | ENSMUST00000022380 | Psmc6 | 14 | ENSG00000100519 | proteasome (prosome, macropain) 26S subunit, ATPase, 6 Gene |  | |
|  | ENSMUST00000022849 | Tars | 15 | ENSG00000113407 | threonyl-tRNA synthetase Gene |  | |
|  | ENSMUST00000023210 | Cyc1 | 15 | ENSG00000179091 | cytochrome c-1 Gene |  | |
|  | ENSMUST00000023221 | Gpaa1 | 15 | ENSG00000197858 | GPI anchor attachment protein 1 Gene |  | |
|  | ENSMUST00000023238 | Gsdmd | 15 | ENSG00000104518 | gasdermin D Gene |  | |
|  | ENSMUST00000023455 | Ppil2 | 16 | ENSG00000100023 | yippee-like 1 (Drosophila) Gene |  | |
|  | ENSMUST00000023693 | Ifnar2 | 16 | ENSG00000159110 | interferon (alpha and beta) receptor 2 Gene |  | |
|  | ENSMUST00000023911 | Nagpa | 16 | ENSG00000103174 | N-acetylglucosamine-1-phosphodiester alpha-N-acetylglucosaminidase Gene |  | |
|  | ENSMUST00000023918 | Ivns1abp | 1 | ENSG00000116679 | influenza virus NS1A binding protein Gene |  | |
|  | ENSMUST00000024206 | Gnb3 | 6 | ENSG00000111664 | guanine nucleotide binding protein (G protein), beta 3 Gene |  | |
|  | ENSMUST00000024486 | Mrps23 | 11 | ENSG00000181610 | mitochondrial ribosomal protein S23 Gene |  | |
|  | ENSMUST00000024976 | Spsb3 | 17 | ENSG00000162032 | splA/ryanodine receptor domain and SOCS box containing 3 Gene |  | |
|  | ENSMUST00000024978 | Nme3 | 17 | ENSG00000103024 | non-metastatic cells 3, protein expressed in Gene |  | |
|  | ENSMUST00000025027 | Cuta | 17 | ENSG00000112514 | cutA divalent cation tolerance homolog (E. coli) Gene |  | |
|  | ENSMUST00000025137 | Thoc1 | 18 | ENSG00000079134 | THO complex 1 Gene |  | |
|  | ENSMUST00000025170 | Wdr46 | 17 | ENSG00000204221 | WD repeat domain 46 Gene |  | |
|  | ENSMUST00000025385 | Hsd17b4 | 18 | ENSG00000133835 | hydroxysteroid (17-beta) dehydrogenase 4 Gene |  | |
|  | ENSMUST00000025547 | 1700034H14Rik | 18 | ENSG00000075336 | RIKEN cDNA 1700034H14 gene Gene |  | |
|  | ENSMUST00000025713 | Tm7sf2 | 19 | ENSG00000149809 | transmembrane 7 superfamily member 2 Gene |  | |
|  | ENSMUST00000025841 | Mus81 | 19 | ENSG00000172732 | MUS81 endonuclease homolog (yeast) Gene |  | |
|  | ENSMUST00000025846 | Saps3 | 19 | ENSG00000110075 | SAPS domain family, member 3 Gene |  | |
|  | ENSMUST00000025885 | Sssca1 | 19 | ENSG00000173465 | Sjogren's syndrome/scleroderma autoantigen 1 homolog (human) Gene |  | |
|  | ENSMUST00000026144 | Dcxr | 11 | ENSG00000169738 | dicarbonyl L-xylulose reductase Gene |  | |
|  | ENSMUST00000026318 | Sat1 | X | ENSG00000130066 | solute carrier family 26 (sulfate transporter), member 1 Gene |  | |
|  | ENSMUST00000026479 | Dctn2 | 10 | ENSG00000175203 | dynactin 2 Gene |  | |
|  | ENSMUST00000026832 | 2610003J06Rik | 17 | ENSG00000161999 | jumonji domain containing 8 Gene |  | |
|  | ENSMUST00000026833 | Wdr24 | 17 | ENSG00000127580 | WD repeat domain 24 Gene |  | |
|  | ENSMUST00000026987 | D13Wsu177e | 13 | ENSG00000048162 | UPF0384 protein CGI-117 homolog |  | |
|  | ENSMUST00000027251 | Rev1 | 1 | ENSG00000135945 | REV1 homolog (S. cerevisiae) Gene |  | |
|  | ENSMUST00000027384 | Atic | 1 | ENSG00000138363 | 5-aminoimidazole-4-carboxamide ribonucleotide formyltransferase/IMP cyclohydrolase Gene |  | |
|  | ENSMUST00000027488 | Capn10 | 1 | ENSG00000142330 | calpain 10 Gene |  | |
|  | ENSMUST00000027587 | Ccnt2 | 1 | ENSG00000082258 | cyclin T2 Gene |  | |
|  | ENSMUST00000028342 | Ssna1 | 2 | ENSG00000176101 | Sjogren's syndrome nuclear autoantigen 1 Gene |  | |
|  | ENSMUST00000028349 | Arrdc1 | 2 | ENSG00000197070 | arrestin domain containing 1 Gene |  | |
|  | ENSMUST00000028554 | Agpat7 | 2 | ENSG00000176454 | lysophosphatidylcholine acyltransferase 4 Gene |  | |
|  | ENSMUST00000028599 | Cstf3 | 2 | ENSG00000176102 | cleavage stimulation factor, 3' pre-RNA, subunit 3 Gene |  | |
|  | ENSMUST00000028769 | Ptpra | 2 | ENSG00000132670 | protein tyrosine phosphatase, receptor type, A Gene |  | |
|  | ENSMUST00000028783 | Spint1 | 2 | ENSG00000166145 | serine protease inhibitor, Kunitz type 1 Gene |  | |
|  | ENSMUST00000028892 | Idh3b | 2 | ENSG00000101365 | isocitrate dehydrogenase 3 (NAD+) beta Gene |  | |
|  | ENSMUST00000028900 | Vps16 | 2 | ENSG00000215305 | vacuolar protein sorting 16 (yeast) Gene |  | |
|  | ENSMUST00000028914 | Polr3f | 2 | ENSG00000132664 | polymerase (RNA) III (DNA directed) polypeptide F Gene |  | |
|  | ENSMUST00000029490 | Ahcyl1 | 3 | ENSG00000168710 | S-adenosylhomocysteine hydrolase-like 1 Gene |  | |
|  | ENSMUST00000029563 | Adar | 3 | ENSG00000160710 | adenosine deaminase, RNA-specific Gene |  | |
|  | ENSMUST00000029682 | Thbs3 | 3 | ENSG00000169231 | thrombospondin 3 Gene |  | |
|  | ENSMUST00000029694 | Arhgef2 | 3 | ENSG00000116584 | rho/rac guanine nucleotide exchange factor (GEF) 2 Gene |  | |
|  | ENSMUST00000029910 | Nsmaf | 4 | ENSG00000035681 | neutral sphingomyelinase (N-SMase) activation associated factor Gene |  | |
|  | ENSMUST00000030165 | Fancg | 4 | ENSG00000221829 | Fanconi anemia, complementation group G Gene |  | |
|  | ENSMUST00000030677 | Map3k6 | 4 | ENSG00000142733 | mitogen-activated protein kinase kinase kinase 6 Gene |  | |
|  | ENSMUST00000030800 | Fastk | 5 | ENSG00000164896 | Fas-activated serine/threonine kinase Gene |  | |
|  | ENSMUST00000030901 | Cpsf3l | 4 | ENSG00000127054 | cleavage and polyadenylation specific factor 3-like Gene |  | |
|  | ENSMUST00000031034 | Nrbp1 | 5 | ENSG00000115216 | nuclear receptor binding protein 1 Gene |  | |
|  | ENSMUST00000031271 | Hnrpdl | 5 | ENSG00000152795 | heterogeneous nuclear ribonucleoprotein D-like Gene |  | |
|  | ENSMUST00000031625 | Arpc1a | 5 | ENSG00000013455 | actin related protein 2/3 complex, subunit 1A Gene |  | |
|  | ENSMUST00000031766 | Asns | 6 | ENSG00000070669 | asparagine synthetase Gene |  | |
|  | ENSMUST00000032726 | Tm2d3 | 7 | ENSG00000184277 | TM2 domain containing 3 Gene |  | |
|  | ENSMUST00000033093 | Bax | 7 | ENSG00000087088 | BCL2-associated X protein Gene |  | |
|  | ENSMUST00000033095 | Prr14 | 7 | ENSG00000156858 | proline rich 14 Gene |  | |
|  | ENSMUST00000033652 | Phka2 | X | ENSG00000044446 | phosphorylase kinase alpha 2 Gene |  | |
|  | ENSMUST00000033939 | Ikbkb | 8 | ENSG00000104365 | inhibitor of kappaB kinase beta Gene |  | |
|  | ENSMUST00000034121 | Man2b1 | 8 | ENSG00000104774 | mannosidase 2, alpha B1 Gene |  | |
|  | ENSMUST00000034369 | Psmb10 | 8 | ENSG00000205220 | proteasome (prosome, macropain) subunit, beta type 10 Gene |  | |
|  | ENSMUST00000034543 | Rpusd4 | 9 | ENSG00000165526 | RNA pseudouridylate synthase domain containing 4 Gene |  | |
|  | ENSMUST00000034623 | Trappc4 | 9 | ENSG00000196655 | trafficking protein particle complex 4 Gene |  | |
|  | ENSMUST00000034834 | Pkm2 | 9 | ENSG00000067225 | pyruvate kinase, muscle Gene |  | |
|  | ENSMUST00000034987 | Dopey1 | 9 | ENSG00000083097 | dopey family member 1 Gene |  | |
|  | ENSMUST00000035230 | Amt | 9 | ENSG00000145020 | aminomethyltransferase Gene |  | |
|  | ENSMUST00000035242 | Rab24 | 13 | ENSG00000169228 | RAB24, member RAS oncogene family Gene |  | |
|  | ENSMUST00000035481 | Chchd5 | 2 | ENSG00000125611 | coiled-coil-helix-coiled-coil-helix domain containing 5 Gene |  | |
|  | ENSMUST00000035532 | Pik3r1 | 13 | ENSG00000145675 | phosphatidylinositol 3-kinase, regulatory subunit, polypeptide 1 (p85 alpha) Gene |  | |
|  | ENSMUST00000035797 | Rab26 | 17 | ENSG00000167964 | RAB26, member RAS oncogene family Gene |  | |
|  | ENSMUST00000035925 | Slc7a6os | 8 | ENSG00000103061 | solute carrier family 7, member 6 opposite strand Gene |  | |
|  | ENSMUST00000036380 | Atp6v0b | 4 | ENSG00000117410 | ATPase, H+ transporting, lysosomal V0 subunit B Gene |  | |
|  | ENSMUST00000037001 | Letmd1 | 15 | ENSG00000050426 | LETM1 domain containing 1 Gene |  | |
|  | ENSMUST00000037376 | Nagk | 6 | ENSG00000124357 | N-acetylglucosamine kinase Gene |  | |
|  | ENSMUST00000038859 | Pik3cd | 4 | ENSG00000171608 | phosphatidylinositol 3-kinase catalytic delta polypeptide Gene |  | |
|  | ENSMUST00000040518 | Eif3eip | 15 | ENSG00000100129 | eukaryotic translation initiation factor 3, subunit E interacting protein Gene |  | |
|  | ENSMUST00000040776 | Cenpt | 8 | ENSG00000102901 | centromere protein T Gene |  | |
|  | ENSMUST00000041367 | BC057552 | 8 | ENSG00000132017 | DDB1- and CUL4-associated factor 15 |  | |
|  | ENSMUST00000041385 | Arhgap27 | 11 | ENSG00000185602 | Rho GTPase activating protein 27 Gene |  | |
|  | ENSMUST00000042121 | H2-DMa | 17 | ENSG00000204257 | histocompatibility 2, class II, locus DMa Gene |  | |
|  | ENSMUST00000042235 | Eef1a1 | 9 | ENSG00000156508 | eukaryotic translation elongation factor 1 alpha 1 Gene |  | |
|  | ENSMUST00000042412 | Hey1 | 3 | ENSG00000164683 | hairy/enhancer-of-split related with YRPW motif 1 Gene |  | |
|  | ENSMUST00000042498 | Hdlbp | 1 | ENSG00000115677 | high density lipoprotein (HDL) binding protein Gene |  | |
|  | ENSMUST00000042506 | Sgsm3 | 15 | ENSG00000100359 | small G protein signaling modulator 3 Gene |  | |
|  | ENSMUST00000042608 | Acd | 8 | ENSG00000102977 | adrenocortical dysplasia Gene |  | |
|  | ENSMUST00000043269 | Hnrnpk | 13 | ENSG00000165119 | heterogeneous nuclear ribonucleoprotein K Gene |  | |
|  | ENSMUST00000043286 | Poli | 18 | ENSG00000101751 | polymerase (DNA directed), iota Gene |  | |
|  | ENSMUST00000043531 | 2310066E14Rik | 8 | ENSG00000039523 | RIKEN cDNA 2310066E14 gene Gene |  | |
|  | ENSMUST00000043654 | Tubg2 | 11 | ENSG00000037042 | tubulin, gamma 2 Gene |  | |
|  | ENSMUST00000043707 | Rhbdd2 | 5 | ENSG00000005486 | rhomboid domain containing 2 Gene |  | |
|  | ENSMUST00000045110 | Ripk5 | 1 | ENSG00000133059 | receptor interacting protein kinase 5 Gene |  | |
|  | ENSMUST00000045295 | Pnpla7 | 2 | ENSG00000130653 | patatin-like phospholipase domain containing 7 Gene |  | |
|  | ENSMUST00000045633 | Mybbp1a | 11 | ENSG00000132382 | MYB binding protein (P160) 1a Gene |  | |
|  | ENSMUST00000045697 | Mrpl55 | 11 | ENSG00000162910 | mitochondrial ribosomal protein L55 Gene |  | |
|  | ENSMUST00000045737 | Galnt11 | 5 | ENSG00000178234 | UDP-N-acetyl-alpha-D-galactosamine:polypeptide N-acetylgalactosaminyltransferase 11 Gene |  | |
|  | ENSMUST00000045807 | Tsr1 | 11 | ENSG00000167721 | TSR1, 20S rRNA accumulation, homolog (yeast) Gene |  | |
|  | ENSMUST00000046260 | 230696 | 4 | ENSG00000164008 | Uncharacterized protein C1orf50 homolog |  | |
|  | ENSMUST00000046575 | Ptov1 | 7 | ENSG00000104960 | prostate tumor over expressed gene 1 Gene |  | |
|  | ENSMUST00000047226 | Lonp1 | 17 | ENSG00000196365 | lon peptidase 1, mitochondrial Gene |  | |
|  | ENSMUST00000047865 | Mbp | 18 | ENSG00000197971 | myelin basic protein Gene |  | |
|  | ENSMUST00000049353 | Zfp692 | 11 | ENSG00000171163 | zinc finger protein 692 Gene |  | |
|  | ENSMUST00000049382 | Gatad2b | 3 | ENSG00000143614 | GATA zinc finger domain containing 2B Gene |  | |
|  | ENSMUST00000049424 | Wdr74 | 19 | ENSG00000133316 | WD repeat domain 74 Gene |  | |
|  | ENSMUST00000049460 | Grn | 11 | ENSG00000030582 | granulin Gene |  | |
|  | ENSMUST00000050611 | Cep68 | 11 | ENSG00000011523 | centrosomal protein 68 Gene |  | |
|  | ENSMUST00000051512 | Wdr81 | 11 | ENSG00000167716 | WD repeat domain 81 Gene |  | |
|  | ENSMUST00000051822 | Wdr61 | 9 | ENSG00000140395 | WD repeat domain 61 Gene |  | |
|  | ENSMUST00000052332 | Abi2 | 1 | ENSG00000138443 | abl-interactor 2 Gene |  | |
|  | ENSMUST00000052566 | Tmem199 | 11 | ENSG00000160629 | transmembrane protein 199 Gene |  | |
|  | ENSMUST00000052965 | Nipbl | 15 | ENSG00000164190 | Nipped-B homolog (Drosophila) Gene |  | |
|  | ENSMUST00000053131 | Ncam1 | 9 | ENSG00000149294 | neural cell adhesion molecule 1 Gene |  | |
|  | ENSMUST00000053230 | Ulk3 | 9 | ENSG00000140474 | unc-51-like kinase 3 (C. elegans) Gene |  | |
|  | ENSMUST00000053264 | 2410089E03Rik | 15 | ENSG00000197603 | RIKEN cDNA 2410089E03 gene Gene |  | |
|  | ENSMUST00000055506 | Gtf3c1 | 7 | ENSG00000077235 | general transcription factor III C 1 Gene |  | |
|  | ENSMUST00000056034 | 2610110G12Rik | 17 | ENSG00000137343 | RIKEN cDNA 2610110G12 gene Gene |  | |
|  | ENSMUST00000056370 | Pmf1 | 3 | ENSG00000160783 | polyamine-modulated factor 1 Gene |  | |
|  | ENSMUST00000058639 | Gm71 | 12 | ENSG00000100483 | gene model 71, (NCBI) Gene |  | |
|  | ENSMUST00000060808 | Plxnb2 | 15 | ENSG00000196576 | plexin B2 Gene |  | |
|  | ENSMUST00000060834 | Alkbh6 | 7 | ENSG00000221889 | alkB, alkylation repair homolog 6 (E. coli) Gene |  | |
|  | ENSMUST00000062193 | Tpm3 | 3 | ENSG00000143549 | tropomyosin 3, gamma Gene |  | |
|  | ENSMUST00000063344 | Tmem112 | 17 | ENSG00000103227 | lipase maturation factor 1 Gene |  | |
|  | ENSMUST00000063761 | Cpt1c | 7 | ENSG00000169169 | carnitine palmitoyltransferase 1c Gene |  | |
|  | ENSMUST00000064454 | Gcn1l1 | 5 | ENSG00000089154 | GCN1 general control of amino-acid synthesis 1-like 1 (yeast) Gene |  | |
|  | ENSMUST00000065014 | Lamb2 | 9 | ENSG00000172037 | laminin, beta 2 Gene |  | |
|  | ENSMUST00000065302 | Cenpj | 14 | ENSG00000151849 | centromere protein J Gene |  | |
|  | ENSMUST00000065330 | Clk3 | 9 | ENSG00000179335 | CDC-like kinase 3 Gene |  | |
|  | ENSMUST00000066587 | Acox1 | 11 | ENSG00000161533 | acyl-Coenzyme A oxidase 1, palmitoyl Gene |  | |
|  | ENSMUST00000066668 | Dnpep | 1 | ENSG00000123992 | aspartyl aminopeptidase Gene |  | |
|  | ENSMUST00000068282 | Arl6ip2 | 17 | ENSG00000119787 | atlastin GTPase 2 Gene |  | |
|  | ENSMUST00000068916 | Ppapdc1b | 8 | ENSG00000147535 | phosphatidic acid phosphatase type 2 domain containing 1B Gene |  | |
|  | ENSMUST00000069064 | Ydjc | 16 | ENSG00000161179 | YdjC homolog (bacterial) Gene |  | |
|  | ENSMUST00000069304 | Hnrph1 | 11 | ENSG00000169045 | heterogeneous nuclear ribonucleoprotein H1 Gene |  | |
|  | ENSMUST00000069318 | Rabggtb | 3 | ENSG00000137955 | RAB geranylgeranyl transferase, b subunit Gene |  | |
|  | ENSMUST00000069530 | Xrcc6 | 15 | ENSG00000196419 | X-ray repair complementing defective repair in Chinese hamster cells 6 Gene |  | |
|  | ENSMUST00000069722 | Taz | X | ENSG00000102125 | tafazzin Gene |  | |
|  | ENSMUST00000070004 | Ldhd | 8 | ENSG00000166816 | lactate dehydrogenase D Gene |  | |
|  | ENSMUST00000071402 | Elovl6 | 3 | ENSG00000170522 | ELOVL family member 6, elongation of long chain fatty acids (yeast) Gene |  | |
|  | ENSMUST00000071898 | Cpsf1 | 15 | ENSG00000071894 | cleavage and polyadenylation specific factor 1 Gene |  | |
|  | ENSMUST00000071926 | Nol7 | 13 | ENSG00000137420 | nucleolar protein 7 Gene |  | |
|  | ENSMUST00000073236 | Ankzf1 | 1 | ENSG00000163516 | ankyrin repeat and zinc finger domain containing 1 Gene |  | |
|  | ENSMUST00000073428 | Slc39a4 | 15 | ENSG00000147804 | solute carrier family 39 (zinc transporter), member 4 Gene |  | |
|  | ENSMUST00000074669 | Hnrpab | 11 | ENSG00000197451 | heterogeneous nuclear ribonucleoprotein A/B Gene |  | |
|  | ENSMUST00000074840 | Preb | 5 | ENSG00000138073 | prolactin regulatory element binding Gene |  | |
|  | ENSMUST00000075406 | BC059842 | 4 | ENSG00000198198 | Novel protein |  | |
|  | ENSMUST00000075856 | Fbxl11 | 19 | ENSG00000173120 | F-box and leucine-rich repeat protein 11 Gene |  | |
|  | ENSMUST00000076493 | Slc22a21 | 11 | ENSG00000197375 | solute carrier family 22 (organic cation transporter), member 21 Gene |  | |
|  | ENSMUST00000076921 | Arl16 | 11 | ENSG00000214087 | ADP-ribosylation factor-like 16 Gene |  | |
|  | ENSMUST00000077353 | Hmbs | 9 | ENSG00000149397 | hydroxymethylbilane synthase Gene |  | |
|  | ENSMUST00000077876 | Snx12 | X | ENSG00000147164 | sorting nexin 12 Gene |  | |
|  | ENSMUST00000077879 | Vps13c | 9 | ENSG00000129003 | vacuolar protein sorting 13C (yeast) Gene |  | |
|  | ENSMUST00000078665 | Dhps | 8 | ENSG00000095059 | deoxyhypusine synthase Gene |  | |
|  | ENSMUST00000081314 | Blm | 7 | ENSG00000197299 | Bloom syndrome homolog (human) Gene |  | |
|  | ENSMUST00000081318 | Sfi1 | 11 | ENSG00000198089 | Sfi1 homolog, spindle assembly associated (yeast) Gene |  | |
|  | ENSMUST00000082177 | Jarid1c | X | ENSG00000126012 | jumonji, AT rich interactive domain 1C (Rbp2 like) Gene |  | |
|  | ENSMUST00000082223 | Rpl5 | 5 | ENSG00000122406 | ribosomal protein L5 Gene |  | |
|  | ENSMUST00000084985 | Ppp2r5c | 12 | ENSG00000078304 | protein phosphatase 2, regulatory subunit B (B56), gamma isoform Gene |  | |
|  | ENSMUST00000085272 | Htatip2 | 7 | ENSG00000109854 | HIV-1 tat interactive protein 2, homolog (human) Gene |  | |
|  | ENSMUST00000085358 | Tex9 | 9 | ENSG00000151575 | testis expressed gene 9 Gene |  | |
|  | ENSMUST00000085835 | Map4k1 | 7 | ENSG00000104814 | mitogen-activated protein kinase kinase kinase kinase 1 Gene |  | |
|  | ENSMUST00000086199 | Glul | 1 | ENSG00000135821 | glutamate-ammonia ligase (glutamine synthetase) Gene |  | |
|  | ENSMUST00000086216 | Anapc5 | 5 | ENSG00000089053 | anaphase-promoting complex subunit 5 Gene |  | |
|  | ENSMUST00000087122 | Speg | 1 | ENSG00000072195 | SPEG complex locus Gene |  | |
|  | ENSMUST00000087315 | Vars | 17 | ENSG00000204394 | valyl-tRNA synthetase Gene |  | |
|  | ENSMUST00000088345 | Mapk8ip3 | 17 | ENSG00000138834 | mitogen-activated protein kinase 8 interacting protein 3 Gene |  | |
|  | ENSMUST00000089024 | Tcp1 | 17 | ENSG00000120438 | t-complex protein 1 Gene |  | |
|  | ENSMUST00000089581 | A930025D01Rik | 2 | ENSG00000132635 | family with sequence similarity 113, member A Gene |  | |
|  | ENSMUST00000090558 | Celsr2 | 3 | ENSG00000143126 | cadherin, EGF LAG seven-pass G-type receptor 2 (flamingo homolog, Drosophila) Gene |  | |
|  | ENSMUST00000090561 | Psrc1 | 3 | ENSG00000134222 | proline/serine-rich coiled-coil 1 Gene |  | |
|  | ENSMUST00000090927 | Clk2 | 3 | ENSG00000176444 | CDC-like kinase 2 Gene |  | |
|  | ENSMUST00000090941 | Msto1 | 3 | ENSG00000125459 | misato homolog 1 (Drosophila) Gene |  | |
|  | ENSMUST00000092324 | Ptbp1 | 10 | ENSG00000011304 | polypyrimidine tract binding protein 1 Gene |  | |
|  | ENSMUST00000092887 | Myo18a | 11 | ENSG00000196535 | myosin XVIIIa Gene |  | |
|  | ENSMUST00000093193 | Dock2 | 11 | ENSG00000134516 | dedicator of cyto-kinesis 2 Gene |  | |
|  | ENSMUST00000093211 | Elmo3 | 8 | ENSG00000102890 | engulfment and cell motility 3, ced-12 homolog (C. elegans) Gene |  | |
|  | ENSMUST00000093468 | Psd3 | 8 | ENSG00000156011 | proteasome (prosome, macropain) 26S subunit, non-ATPase, 3 Gene |  | |
|  | ENSMUST00000095426 | AC152410.6 | 10 | ENSG00000099840 |  | | |
|  | ENSMUST00000095775 | Setdb2 | 14 | ENSG00000136169 | SET domain, bifurcated 2 Gene | |  |
|  | ENSMUST00000095806 | Map3k5 | 10 | ENSG00000197442 | mitogen-activated protein kinase kinase kinase 5 Gene | |  |
|  | ENSMUST00000096255 | Ubxn1 | 19 | ENSG00000162191 | UBX domain protein 1 Gene | |  |
|  | ENSMUST00000097373 | Tsc2 | 17 | ENSG00000103197 | tuberous sclerosis 2 Gene | |  |
|  | ENSMUST00000097737 | Pusl1 | 4 | ENSG00000169972 | pseudouridylate synthase-like 1 Gene | |  |
|  | ENSMUST00000098461 | Cd37 | 7 | ENSG00000104894 | CD37 antigen Gene | |  |
|  | ENSMUST00000100143 | Rc3h2 | 2 | ENSG00000056586 | ring finger and CCCH-type zinc finger domains 2 Gene | |  |
|  | ENSMUST00000102574 | Acadvl | 11 | ENSG00000072778 | acyl-Coenzyme A dehydrogenase, very long chain Gene | |  |
|  | ENSMUST00000102589 | Eif4a1 | 11 | ENSG00000161960 | eukaryotic translation initiation factor 4A1 Gene | |  |
|  | ENSMUST00000102702 | Guk1 | 11 | ENSG00000143774 | guanylate kinase 1 Gene | |  |
|  | ENSMUST00000103128 | Rpn2 | 2 | ENSG00000118705 | ribophorin II Gene | |  |
|  | ENSMUST00000103198 | Nol5a | 2 | ENSG00000101361 | nucleolar protein 5A Gene | |  |
|  | ENSMUST00000106226 | Tial1 | 7 | ENSG00000151923 | Tia1 cytotoxic granule-associated RNA binding protein-like 1 Gene | |  |
|  | ENSMUST00000106908 | Pde4b | 4 | ENSG00000184588 | phosphodiesterase 4B, cAMP specific Gene | |  |
|  | ENSMUST00000107384 | Idh2 | 7 | ENSG00000182054 | isocitrate dehydrogenase 2 (NADP+), mitochondrial Gene | |  |
|  | ENSMUST00000108627 | Tsen34 | 7 | ENSG00000170892 | tRNA splicing endonuclease 34 homolog (S. cerevisiae) Gene | |  |
|  | ENSMUST00000109075 | Th1l | 2 | ENSG00000101158 | TH1-like homolog (Drosophila) Gene | |  |
|  | ENSMUST00000109324 | Sbf1 | 15 | ENSG00000100241 | SET binding factor 1 Gene | |  |
|  | ENSMUST00000110623 | Fgfr1 | 8 | ENSG00000077782 | fibroblast growth factor receptor 1 Gene | |  |
|  | ENSMUST00000111305 | Usp21 | 1 | ENSG00000143258 | ubiquitin specific peptidase 21 Gene | |  |
|  | ENSMUST00000113707 | Tpm1 | 9 | ENSG00000140416 | T-cell phenotype modifier 1 QTL | |  |
|  | ENSMUST00000113913 | Dctn1 | 6 | ENSG00000204843 | dynactin 1 Gene | |  |
|  | ENSMUST00000114349 | Ndor1 | 2 | ENSG00000188566 | NADPH dependent diflavin oxidoreductase 1 Gene | |  |
|  | ENSMUST00000114512 | Gls | 1 | ENSG00000115419 | glutaminase Gene | |  |
|  | ENSMUST00000114700 | Agbl5 | 5 | ENSG00000084693 | ATP/GTP binding protein-like 5 Gene | |  |
|  | ENSMUST00000115728 | Tmem173 | 18 | ENSG00000184584 | transmembrane protein 173 Gene | |  |
|  | ENSMUST00000116363 | 2310004I24Rik | 11 | ENSG00000170222 | RIKEN cDNA 2310004I24 gene Gene | |  |
|  | ENSMUST00000118163 | Dmxl2 | 9 | ENSG00000104093 | Dmx-like 2 Gene | |  |
|  | ***Rattus norvegicus*** | | | | | |  |
|  | Transcript | Gene Name | Chr | Human ortholog ID | Description | |  |
|  | ENSRNOT00000005915 | Ddx39 | 19 | ENSG00000123136 | ATP-dependent RNA helicase DDX39 (EC 3.6.1.-) (DEAD box protein 39) (Nuclear RNA helicase, DECD variant of DEAD box family). | | |
|  | ENSRNOT00000006137 | Sip1 | 6 | ENSG00000092208 | Survival of motor neuron protein-interacting protein 1 (SMN- interacting protein 1) (Component of gems 2) (Gemin-2). | | |
|  | ENSRNOT00000007103 | NP_001013223.2 | 3 | ENSG00000104177 | myelin basic protein expression factor 2, repressor | | |
|  | ENSRNOT00000009042 | Slc5a6 | 6 | ENSG00000138074 | Sodium-dependent multivitamin transporter (Na(+)-dependent multivitamin transporter) (Solute carrier family 5 member 6). | | |
|  | ENSRNOT00000009564 | Nol5a | 3 | ENSG00000101361 | nucleolar protein 5A | | |
|  | ENSRNOT00000009649 | Psmc6 | 15 | ENSG00000100519 | Psmc6 protein (Fragment). | | |
|  | ENSRNOT00000010467 | NP_001101576.1 | 7 | ENSG00000196576 | plexin B2 | | |
|  | ENSRNOT00000011682 | Rbms1 | 3 | ENSG00000153250 | RNA-binding motif, single-stranded-interacting protein 1. | | |
|  | ENSRNOT00000012463 | NP_001101379.1 | 5 | ENSG00000142733 | mitogen-activated protein kinase kinase kinase 6 | | |
|  | ENSRNOT00000013573 | Prpsap1 | 10 | ENSG00000161542 | Phosphoribosyl pyrophosphate synthetase-associated protein 1 (PRPP synthetase-associated protein 1) (39 kDa phosphoribosypyrophosphate synthetase-associated protein) (PAP39). | | |
|  | ENSRNOT00000015247 | NP_001099549.1 | 16 | ENSG00000105726 | ATPase type 13A1 | | |
|  | ENSRNOT00000015680 | Fst | 2 | ENSG00000134363 | Follistatin precursor (FS) (Activin-binding protein). | | |
|  | ENSRNOT00000017045 | RGD1311805 | 18 | ENSG00000141452 | similar to RIKEN cDNA 2400010D15 (RGD1311805), mRNA | | |
|  | ENSRNOT00000017407 | Unc45a | 1 | ENSG00000140553 | UNC45 homolog A (UNC-45A) (Smooth muscle cell-associated protein 1) (SMAP-1). | | |
|  | ENSRNOT00000017722 | NP_001102263.1 | 9 | ENSG00000136710 | | | |
|  | ENSRNOT00000017942 | C12orf10 | 7 | ENSG00000139637 | MYG1 protein | | |
|  | ENSRNOT00000019886 | Galt | 5 | ENSG00000213930 | Galactose-1-phosphate uridylyltransferase (EC 2.7.7.12) (Gal-1-P uridylyltransferase) (UDP-glucose--hexose-1-phosphate uridylyltransferase). | | |
|  | ENSRNOT00000020839 | Ssx2ip | 2 | ENSG00000117155 | Afadin- and alpha-actinin-binding protein (ADIP) (Afadin DIL domain- interacting protein). | | |
|  | ENSRNOT00000022275 | Gstk1 | 4 | ENSG00000197448 | Glutathione S-transferase kappa 1 (EC 2.5.1.18) (GST 13-13) (Glutathione S-transferase subunit 13) (GST class-kappa) (GSTK1-1) (rGSTK1). | | |
|  | ENSRNOT00000022486 | SUV41_RAT | 1 | ENSG00000110066 | Histone-lysine N-methyltransferase SUV420H1 (EC 2.1.1.43) (Suppressor of variegation 4-20 homolog 1) (Suv4-20h1) (Su(var)4-20 homolog 1). | | |
|  | ENSRNOT00000022650 | RGD1306660 | 16 | ENSG00000053501 | | | |
|  | ENSRNOT00000024440 | NP_001101703.1 | 9 | ENSG00000006607 | FERM, RhoGEF and pleckstrin domain protein 2 | | |
|  | ENSRNOT00000025196 | Slc3a2 | 1 | ENSG00000168003 | 4F2 cell-surface antigen heavy chain (4F2hc). | | |
|  | ENSRNOT00000025315 | Ggtl3 | 3 | ENSG00000131067 | Gamma-glutamyltransferase 4 precursor (EC 2.3.2.2) (Gamma- glutamyltranspeptidase 4) (GGT 4) (Gamma-glutamyltransferase-like 3) | | |
|  | ENSRNOT00000025906 | Ilk | 1 | ENSG00000166333 | Integrin-linked protein kinase (EC 2.7.11.1). | | |
|  | ENSRNOT00000026111 | Sec16a | 3 | ENSG00000148396 | SEC16 homolog A (S. cerevisiae) Gene | | |
|  | ENSRNOT00000026641 | RGD1306126 | 10 | ENSG00000103254 | RGD1306126 protein. | | |
|  | ENSRNOT00000026725 | RGD1306841 | 5 | ENSG00000127054 | Integrator complex subunit 11 (EC 3.1.27.-) (Int11) (Cleavage and polyadenylation-specific factor 3-like protein) (CPSF3-like protein). | | |
|  | ENSRNOT00000027263 | Celsr2 | 2 | ENSG00000143126 | Cadherin EGF LAG seven-pass G-type receptor 2 (Multiple epidermal growth factor-like domains 3) (Fragment). | | |
|  | ENSRNOT00000027580 | Ptov1 | 1 | ENSG00000104960 | Prostate tumor overexpressed gene 1 protein homolog. | | |
|  | ENSRNOT00000027944 | Fcgrt | 1 | ENSG00000104870 | IgG receptor FcRn large subunit p51 precursor (FcRn) (Neonatal Fc receptor) (IgG Fc fragment receptor transporter alpha chain). | | |
|  | ENSRNOT00000028793 | Txnip | 2 | ENSG00000117289 | Thioredoxin-interacting protein (Vitamin D3 up-regulated protein 1). | | |
|  | ENSRNOT00000030934 | NP_001100383.1 | 9 | ENSG00000013441 | CDC-like kinase 1 | | |
|  | ENSRNOT00000034386 | Nob1p | 19 | ENSG00000141101 | RNA-binding protein NOB1. | | |
|  | ENSRNOT00000037160 | LOC684910 | 5 | ENSG00000066322 | similar to Elongation of very long chain fatty acids protein 1 (LOC679532), mRNA | | |
|  | ENSRNOT00000037699 | Man2b1 | 19 | ENSG00000104774 | mannosidase 2, alpha B1 | | |
|  | ENSRNOT00000040541 | Ecgf1 | 7 | ENSG00000025708 | endothelial cell growth factor 1 (platelet-derived) | | |
|  | ENSRNOT00000046456 | Mta1 | 6 | ENSG00000182979 | Metastasis-associated protein MTA1. | | |
|  | ENSRNOT00000049706 | NP_001102224.1 | 8 | ENSG00000105364 | mitochondrial ribosomal protein L4 | | |
|  | ENSRNOT00000055810 | RGD1308836 | 3 | ENSG00000132635 | similar to chromosome 20 open reading frame 81 (RGD1308836), mRNA | | |
|  | ENSRNOT00000055865 | Hdac10 | 7 | ENSG00000100429 | Histone deacetylase 10 (HD10). | | |
|  | ENSRNOT00000056295 | Gdi1 | X | ENSG00000203879 | Rab GDP dissociation inhibitor alpha (Rab GDI alpha) (Guanosine diphosphate dissociation inhibitor 1) (GDI-1). | | |
|  | ENSRNOT00000056397 | Plxnb3_predicted | X | ENSG00000198753 | | | |
|  | ENSRNOT00000057144 | Tbrg4 | 14 | ENSG00000136270 | Protein TBRG4 (Transforming growth factor beta regulator 4). | | |
|  | ENSRNOT00000059187 | RGD1310311 | 6 | ENSG00000165506 | similar to chromosome 14 open reading frame 104 (RGD1310311), mRNA | | |
|  | ***Bos Taurus*** | | | | | | |
|  | Transcript | Gene Name | Chr | Human ortholog ID | Description | | |
|  | ENSBTAT00000000033 | IPI00702306.2 | 19 | ENSG00000187456 | | | |
| ENSBTAT00000000301 | | A6QNY4_BOVIN | 13 | ENSG00000124214 | STAU1 protein Fragment | | |
| ENSBTAT00000000348 | | RAB26_BOVIN | 25 | ENSG00000167964 | Ras-related protein Rab-26 | | |
| ENSBTAT00000001034 | | HS90B_BOVIN | 23 | ENSG00000096384 | Heat shock protein HSP 90-beta | | |
| ENSBTAT00000001696 | | NP_001095561.1 | 18 | ENSG00000102890 | engulfment and cell motility 3 | | |
| ENSBTAT00000001861 | | RM04_BOVIN | 7 | ENSG00000105364 | 39S ribosomal protein L4, mitochondrial (L4mt)(MRP-L4) | | |
| ENSBTAT00000002231 | | NP_001077126.1 | 19 | ENSG00000179029 | transmembrane protein 107 | | |
| ENSBTAT00000003544 | | CP013_BOVIN | 25 | ENSG00000130731 | UPF0585 protein C16orf13 homolog | | |
| ENSBTAT00000003557 | | OSGEP_BOVIN | 10 | ENSG00000092094 | Probable O-sialoglycoprotein endopeptidase (EC 3.4.24.57) | | |
| ENSBTAT00000003561 | | NP_001069827.1 | 10 | ENSG00000165782 | transmembrane protein 55B | | |
| ENSBTAT00000003736 | | AP4M1_BOVIN | 25 | ENSG00000221838 | AP-4 complex subunit mu-1 (Adapter-related protein complex 4 mu-1 subunit)(AP-4 adapter complex mu subunit)(Mu subunit of AP-4)(Mu4-adaptin)(mu4)(Mu-adaptin-related protein 2)(mu-ARP2) | | |
| ENSBTAT00000004442 | | NP_001068713.1 | 13 | ENSG00000125871 | hypothetical protein LOC506205 | | |
| ENSBTAT00000004789 | | LPHN1_BOVIN | 7 | ENSG00000072071 | Latrophilin-1 Precursor (Calcium-independent alpha-latrotoxin receptor 1) | | |
| ENSBTAT00000005152 | | FPPS_BOVIN | 3 | ENSG00000160752 | Farnesyl pyrophosphate synthetase (FPP synthetase)(FPS)(Farnesyl diphosphate synthetase) | | |
| ENSBTAT00000005222 | | IPI00702273.3 | 7 | ENSG00000164576 | | | |
| ENSBTAT00000005441 | | NP_001076876.1 | 19 | ENSG00000006282 | spermatogenesis associated 20 | | |
| ENSBTAT00000006183 | | NP_001039834.1 | 7 | ENSG00000050748 | mitogen-activated protein kinase 9 | | |
| ENSBTAT00000006461 | | M2OM_BOVIN | 19 | ENSG00000108528 | Mitochondrial 2-oxoglutarate/malate carrier protein (OGCP)(Solute carrier family 25 member 11) | | |
| ENSBTAT00000006709 | | IPI00718156.3 | 8 | ENSG00000135048 | | | |
| ENSBTAT00000007052 | | HEM3_BOVIN | 15 | ENSG00000149397 | Porphobilinogen deaminase (EC 2.5.1.61)(Hydroxymethylbilane synthase)(HMBS) | | |
| ENSBTAT00000007065 | | GPT_BOVIN | 15 | ENSG00000172269 | UDP-N-acetylglucosamine--dolichyl-phosphate N-acetylglucosaminephosphotransferase (EC 2.7.8.15)(N-acetylglucosamine-1-phosphate transferase)(GlcNAc-1-P transferase)(G1PT)(GPT) | | |
| ENSBTAT00000007338 | | GATM_BOVIN | 10 | ENSG00000171766 | Glycine amidinotransferase, mitochondrial Precursor (EC 2.1.4.1)(L-arginine:glycine amidinotransferase)(Transamidinase)(AT) | | |
| ENSBTAT00000008186 | | IPI00694462.1 | 3 | ENSG00000143624 | | | |
| ENSBTAT00000008411 | | TMM85_BOVIN | 10 | ENSG00000128463 | Transmembrane protein 85 | | |
| ENSBTAT00000008557 | | IPI00704229.2 | 16 | ENSG00000215788 | | | |
| ENSBTAT00000009070 | | IPI00706061.3 | 5 | ENSG00000111077 | | | |
| ENSBTAT00000009208 | | Q1RMK4_BOVIN | 11 | ENSG00000115306 | SPTBN1 protein Fragment | | |
| ENSBTAT00000009303 | | ABHD1_BOVIN | 11 | ENSG00000143994 | Abhydrolase domain-containing protein 1 (EC 3.1.1.-) | | |
| ENSBTAT00000009383 | | Q148L2_BOVIN | 7 | ENSG00000105726 | ATP13A1 protein Fragment | | |
| ENSBTAT00000009469 | | TAGL_BOVIN | 15 | ENSG00000149591 | Transgelin (Smooth muscle protein 22-alpha)(SM22-alpha)(25 kDa F-actin-binding protein) | | |
| ENSBTAT00000009665 | | COMD4_BOVIN | 21 | ENSG00000140365 | COMM domain-containing protein 4 | | |
| ENSBTAT00000010013 | | BBS4_BOVIN | 10 | ENSG00000140463 | Bardet-Biedl syndrome 4 protein homolog | | |
| ENSBTAT00000010487 | | NP_001076887.1 | 13 | ENSG00000125869 | hypothetical protein LOC513094 | | |
| ENSBTAT00000010702 | | NP_001091470.1 | 2 | ENSG00000078098 | fibroblast activation protein, alpha subunit | | |
| ENSBTAT00000010706 | | IPI00727139.3 | 9 | ENSG00000135587 | | | |
| ENSBTAT00000011048 | | NP_001029586.1 | 7 | ENSG00000011132 | amyloid beta (A4) precursor protein-binding, family A, member 3 | | |
| ENSBTAT00000011704 | | IPI00698364.4 | 12 | ENSG00000027001 | | | |
| ENSBTAT00000012145 | | NP_001073236.1 | 3 | ENSG00000143257 | nuclear receptor subfamily 1, group I, member 3 | | |
| ENSBTAT00000012357 | | IPI00734138.1 | 7 | ENSG00000169045 | | | |
| ENSBTAT00000012413 | | NP_001039690.1 | 3 | ENSG00000160781 | progestin and adipoQ receptor family member VI | | |
| ENSBTAT00000012698 | | S7A6O_BOVIN | 18 | ENSG00000103061 | Protein SLC7A6OS (Solute carrier family 7 member 6 opposite strand transcript homolog) | | |
| ENSBTAT00000013077 | | TPM3_BOVIN | 3 | ENSG00000143549 | Tropomyosin alpha-3 chain (Tropomyosin-3)(Gamma-tropomyosin) | | |
| ENSBTAT00000013079 | | RS3A_BOVIN | 17 | ENSG00000145425 | 40S ribosomal protein S3a | | |
| ENSBTAT00000013845 | | UBXN1_BOVIN | 29 | ENSG00000162191 | UBX domain-containing protein 1 (SAPK substrate protein 1) | | |
| ENSBTAT00000014027 | | A4FV10_BOVIN | 4 | ENSG00000158604 | TMED4 protein Fragment | | |
| ENSBTAT00000014744 | | NP_001068606.1 | 11 | ENSG00000115310 | reticulon 4 isoform 1 | | |
| ENSBTAT00000014894 | | ACTN4_BOVIN | 18 | ENSG00000130402 | Alpha-actinin-4 (Non-muscle alpha-actinin 4)(F-actin cross-linking protein) | | |
| ENSBTAT00000015194 | | IPI00703173.3 | 8 | ENSG00000070610 | | | |
| ENSBTAT00000015555 | | IPI00698201.4 | 19 | ENSG00000108469 | | | |
| ENSBTAT00000015772 | | IPI00905810.1 | 3 | ENSG00000159214 | | | |
| ENSBTAT00000015834 | | Q8HYZ3_BOVIN | 10 | ENSG00000100889 | Phosphoenolpyruvate carboxykinase Fragment | | |
| ENSBTAT00000016093 | | GDIA_BOVIN | X | ENSG00000203879 | Rab GDP dissociation inhibitor alpha (Rab GDI alpha)(Guanosine diphosphate dissociation inhibitor 1)(GDI-1)(SMG p25A GDI) | | |
| ENSBTAT00000016937 | | UBC12_BOVIN | 18 | ENSG00000130725 | NEDD8-conjugating enzyme Ubc12 (EC 6.3.2.-)(Ubiquitin-conjugating enzyme E2 M)(NEDD8 protein ligase)(NEDD8 carrier protein) | | |
| ENSBTAT00000017614 | | NP_001019659.1 | 29 | ENSG00000168003 | solute carrier family 3 (activators of dibasic and neutral amino acid transport), member 2 | | |
| ENSBTAT00000017930 | | NP_001039537.1 | 10 | ENSG00000166140 | zinc finger, FYVE domain containing 19 | | |
| ENSBTAT00000018998 | | RHOC_BOVIN | 3 | ENSG00000155366 | Rho-related GTP-binding protein RhoC Precursor | | |
| ENSBTAT00000019318 | | EF1A1_BOVIN | 9 | ENSG00000156508 | Elongation factor 1-alpha 1 (EF-1-alpha-1)(Elongation factor 1 A-1)(eEF1A-1)(Elongation factor Tu)(EF-Tu) | | |
| ENSBTAT00000020770 | | NP_001092492.1 | 19 | ENSG00000132591 | Era-like 1 | | |
| ENSBTAT00000020997 | | NP_001030213.1 | 8 | ENSG00000213930 | galactose-1-phosphate uridylyltransferase isoform 1 | | |
| ENSBTAT00000021254 | | SIKE_BOVIN | 3 | ENSG00000052723 | Suppressor of IKK-epsilon | | |
| ENSBTAT00000022183 | | NP_001039333.1 | 21 | ENSG00000100906 | nuclear factor of kappa light polypeptide gene enhancer in B-cells inhibitor, alpha | | |
| ENSBTAT00000022381 | | NP_001015564.1 | 23 | ENSG00000204221 | WD repeat domain 46 | | |
| ENSBTAT00000022385 | | NP_001068622.1 | 23 | ENSG00000204218 | ral guanine nucleotide dissociation stimulator-like 2 | | |
| ENSBTAT00000023162 | | TM214_BOVIN | 11 | ENSG00000119777 | Transmembrane protein 214 | | |
| ENSBTAT00000023571 | | K0859_BOVIN | 16 | ENSG00000010165 | Putative methyltransferase KIAA0859 homolog (EC 2.1.1.-) | | |
| ENSBTAT00000023751 | | CAV1_BOVIN | 4 | ENSG00000105974 | Caveolin-1 | | |
| ENSBTAT00000024015 | | PSB10_BOVIN | 18 | ENSG00000205220 | Proteasome subunit beta type-10 Precursor (EC 3.4.25.1)(Proteasome subunit beta-2i) | | |
| ENSBTAT00000024316 | | MPV17_BOVIN | 11 | ENSG00000115204 | Protein Mpv17 | | |
| ENSBTAT00000024514 | | Q3SYZ5_BOVIN | 19 | ENSG00000108654 | DDX5 protein Fragment | | |
| ENSBTAT00000025020 | | PGTA_BOVIN | 10 | ENSG00000100949 | Geranylgeranyl transferase type-2 subunit alpha (EC 2.5.1.60)(Geranylgeranyl transferase type II subunit alpha)(Rab geranylgeranyltransferase subunit alpha)(Rab geranyl-geranyltransferase subunit alpha)(Rab GG transferase alpha)(Rab GGTase alpha) | | |
| ENSBTAT00000025031 | | NP_001068679.1 | 19 | ENSG00000159210 | EAP30 subunit of ELL complex | | |
| ENSBTAT00000025033 | | IPI00711984.3 | 3 | ENSG00000143126 | | | |
| ENSBTAT00000025159 | | A6QQ10_BOVIN | 18 | ENSG00000102977 | ACD protein Fragment | | |
| ENSBTAT00000025227 | | IPI00707133.3 | 24 | ENSG00000134759 | | | |
| ENSBTAT00000025243 | | NP_001069687.1 | 23 | ENSG00000137207 | natural killer cell-specific antigen KLIP1 | | |
| ENSBTAT00000025396 | | IPI00713016.1 | 10 | ENSG00000137843 | | | |
| ENSBTAT00000026578 | | ARMC8_BOVIN | 1 | ENSG00000114098 | Armadillo repeat-containing protein 8 | | |
| ENSBTAT00000026698 | | NP_001071369.1 | 10 | ENSG00000176454 | PLSC domain containing protein | | |
| ENSBTAT00000027046 | | NP_001091486.1 | 5 | ENSG00000111679 | protein tyrosine phosphatase, non-receptor type 6 | | |
| ENSBTAT00000027688 | | GPX4_BOVIN | 7 | ENSG00000167468 | Phospholipid hydroperoxide glutathione peroxidase, mitochondrial Precursor (PHGPx)(EC 1.11.1.12)(Glutathione peroxidase 4)(GPx-4) | | |
| ENSBTAT00000028048 | | PRS8_BOVIN | 19 | ENSG00000087191 | 26S protease regulatory subunit 8 (Proteasome 26S subunit ATPase 5)(Proteasome subunit p45)(p45/SUG) | | |
| ENSBTAT00000028350 | | CIB1_BOVIN | 21 | ENSG00000185043 | Calcium and integrin-binding protein 1 (Calmyrin) | | |
| ENSBTAT00000028364 | | PSB4_BOVIN | 3 | ENSG00000159377 | Proteasome subunit beta type-4 Precursor (EC 3.4.25.1) | | |
| ENSBTAT00000028387 | | RSAD1_BOVIN | 19 | ENSG00000136444 | Radical S-adenosyl methionine domain-containing protein 1, mitochondrial Precursor (EC 1.3.99.-)(Oxygen-independent coproporphyrinogen III oxidase-like protein RSAD1) | | |
| ENSBTAT00000028718 | | NP_001029556.1 | 23 | ENSG00000137420 | nucleolar protein 7, 27kDa | | |
| ENSBTAT00000028997 | | Q3SZ14_BOVIN | 3 | ENSG00000081721 | DUSP12 protein Fragment | | |
| ENSBTAT00000029060 | | CQ037_BOVIN | 19 | ENSG00000141741 | Uncharacterized protein C17orf37 homolog | | |
| ENSBTAT00000029075 | | Q148I9_BOVIN | 20 | ENSG00000082196 | AMACR protein Fragment | | |
| ENSBTAT00000029088 | | NP_001029924.1 | 7 | ENSG00000123136 | DEAD (Asp-Glu-Ala-Asp) box polypeptide 39 | | |
| ENSBTAT00000029172 | | CXXC1_BOVIN | 24 | ENSG00000154832 | CpG-binding protein (PHD finger and CXXC domain-containing protein 1)(CXXC-type zinc finger protein 1) | | |
| ENSBTAT00000030181 | | NP_001029668.1 | X | ENSG00000071859 | hypothetical protein LOC515539 | | |
| ENSBTAT00000033827 | | NP_001069749.1 | 11 | ENSG00000130560 | ubiquitin associated domain containing 1 | | |
| ENSBTAT00000035207 | | FXL12_BOVIN | 7 | ENSG00000127452 | F-box/LRR-repeat protein 12 (F-box and leucine-rich repeat protein 12) | | |
| ENSBTAT00000035212 | | NP_001069968.1 | 19 | ENSG00000129197 | RPA interacting protein | | |
| ENSBTAT00000035714 | | NP_001029974.1 | 7 | ENSG00000169228 | RAB24, member RAS oncogene family | | |
| ENSBTAT00000038173 | | ANKZ1_BOVIN | 2 | ENSG00000163516 | Ankyrin repeat and zinc finger domain-containing protein 1 | | |
| ENSBTAT00000038244 | | GLYM_BOVIN | 5 | ENSG00000182199 | Serine hydroxymethyltransferase, mitochondrial Precursor (SHMT)(Serine methylase)(EC 2.1.2.1)(Glycine hydroxymethyltransferase) | | |
| ENSBTAT00000042805 | | NP_001095464.1 | 5 | ENSG00000111181 | solute carrier family 6 (neurotransmitter transporter, betaine/GABA), member 12 | | |
| ENSBTAT00000043510 | | NP_001076071.1 | 7 | ENSG00000183258 | DEAD (Asp-Glu-Ala-Asp) box polypeptide 41 | | |
| ENSBTAT00000045088 | | IPI00725553.2 | 7 | ENSG00000205744 | | | |
| ENSBTAT00000046260 | | NP_001099108.1 | 19 | ENSG00000174231 | PRP8 pre-mRNA processing factor 8 homolog | | |
| ENSBTAT00000046883 | | NP_001069777.1 | X | ENSG00000071889 | family 3, member A protein | | |
| ENSBTAT00000050490 | | KCRB_BOVIN | 21 | ENSG00000166165 | Creatine kinase B-type (EC 2.7.3.2)(Creatine kinase B chain)(B-CK) | | |
| ENSBTAT00000055693 | | RS13_BOVIN | 15 | ENSG00000110700 | 40S ribosomal protein S13 | | |
| ENSBTAT00000055916 | | NP_001094711.1 | 3 | ENSG00000222009 | hypothetical protein LOC615438 | | |
| ENSBTAT00000056722 | | NP_001095757.1 | 8 | ENSG00000221829 | Fanconi anemia, complementation group G | | |
