# Supplementary material for: Large-Scale Evidence for Conservation of NMD Candidature Across Mammals
Source: PLoS One. 2010 Jul 21;5(7):e11695. doi: 10.1371/journal.pone.0011695 (PMC2908137; doi:10.1371/journal.pone.0011695)
Supplement: Table S3 — Summary of the different types of NMD candidate. (0.03 MB DOC) [file pone.0011695.s004.doc]

| **Table S3. Summary events of AS-NMD** | | | | |
| --- | --- | --- | --- | --- |
| Genomes | EST/cDNA supporting the AS | Genes with an AS-NMD candidate | Orthologs* in the human genome | AS-NMD candidates with an orthologous AS-NMD candidate the in human genome** |
| *Homo sapiens*  IR  CSE  ASD  ASA | 2117  10562  1166  932 | 884  444  592  616 | --------  --------  --------  -------- | --------  -------  -------  ------- |
| *Mus musculus*  IR  CSE  ASD  ASA | 725  205  355  443 | 475  137  247  352 | 440  121  229  324 | 153  23  57  72 |
| *Rattus norvegicus*  IR  CSE  ASD  ASA | 123  13  26  42 | 95  10  26  35 | 92  10  26  31 | 32  3  10  12 |
| *Bos Taurus*  IR  CSE  ASD  ASA | 428  32  78  101 | 231  27  49  67 | 211  25  45  62 | 85  9  15  17 |

† Percentage of retained introns whose length is multiple of 3.
