# Supplementary material for: Large-Scale Evidence for Conservation of NMD Candidature Across Mammals
Source: PLoS One. 2010 Jul 21;5(7):e11695. doi: 10.1371/journal.pone.0011695 (PMC2908137; doi:10.1371/journal.pone.0011695)
Supplement: Table S4 — Top five domain types in NMD candidates in the four mammals studied. (0.05 MB DOC) [file pone.0011695.s005.doc]

| Table S4. Top five domains in NMD-candidates | | |
| --- | --- | --- |
| *Homo sapiens* | | |
| pfam | Domain† | description |
| PF00400 | WD-40 repeats | D-repeat proteins are a large family found in all eukaryotes and are implicated in a variety of functions ranging from signal transduction and transcription regulation to cell cycle control and apoptosis. |
| PF00047 | immunoglobulin superfamily | Immunoglobulin-like domains may be involved in protein-protein and protein-ligand interactions. |
| PF00069 | Protein kinases | Transfers the gamma phosphate from nucleotide triphosphates (often ATP) to one or more amino acid residues in a protein substrate side chain, resulting in a conformational change affecting protein function. |
| PF00018 | SH3 (Src homology 3) | often indicative of a protein involved in signal transduction related to cytoskeletal organisation. |
| PF07653 | SH3_2 (Src homology 3) | often indicative of a protein involved in signal transduction related to cytoskeletal organisation. |
|  |  |  |
| *Mus musculus* | | |
| PF00069 | Protein kinases | Transfers the gamma phosphate from nucleotide triphosphates (often ATP) to one or more amino acid residues in a protein substrate side chain, resulting in a conformational change affecting protein function. |
| PF00400 | WD-40 repeats | D-repeat proteins are a large family found in all eukaryotes and are implicated in a variety of functions ranging from signal transduction and transcription regulation to cell cycle control and apoptosis. |
| PF07714 | Protein kinases | transfers the gamma phosphate from nucleotide triphosphates (often ATP) to one or more amino acid residues in a protein substrate side chain, resulting in a conformational change affecting protein function. |
| PF00023 | ankyrin repeat | The repeat has been found in proteins of diverse function such as transcriptional initiators, cell-cycle regulators, cytoskeletal, ion transporters and signal transducers. |
| PF00169 | pleckstrin homology | involved in intracellular signalling or as constituents of the cytoskeleton |
|  |  |  |
| *Rattus norvegicus* | | |
| PF00069 | Protein kinases | Transfers the gamma phosphate from nucleotide triphosphates (often ATP) to one or more amino acid residues in a protein substrate side chain, resulting in a conformational change affecting protein function. |
| PF07714 | Protein kinases | Transfers the gamma phosphate from nucleotide triphosphates (often ATP) to one or more amino acid residues in a protein substrate side chain, resulting in a conformational change affecting protein function. |
| PF01403 | Sema domain | Large family of secreted and transmembrane proteins, some of which function as repellent signals during axon guidance. |
| PF01437 | Plexin repeat | This is a cysteine rich repeat found in several different extracellular receptors. The function of the repeat is unknown. |
| PF01833 | IPT/TIG domain | These domains are found in cell surface receptors such as Met and Ron as well as in intracellular transcription factors where it is involved in DNA binding. |
|  |  |  |
| *Bos taurus* | | |
| PF00271 | DEAD/H helicases | The eukaryotic translation initiation factor 4A (eIF4A) is a member of the DEA(D/H)-box RNA helicase family This is a diverse group of proteins that couples an ATPase activity to RNA binding and unwinding. |
| PF00069 | Protein kinases | transfers the gamma phosphate from nucleotide triphosphates (often ATP) to one or more amino acid residues in a protein substrate side chain, resulting in a conformational change affecting protein function. |
| PF00400 | WD-40 repeats | D-repeat proteins are a large family found in all eukaryotes and are implicated in a variety of functions ranging from signal transduction and transcription regulation to cell cycle control and apoptosis. |
| PF00270 | DEAD and DEAH box helicases | The DEAD box helicases are involved in various aspects of RNA metabolism, including nuclear transcription, pre mRNA splicing, ribosome biogenesis, nucleocytoplasmic transport, translation, RNA decay and organellar gene expression. |
| PF07686 | Immunoglobulin V-set domain | Ig-like domains are involved in a variety of functions, including cell-cell recognition, cell-surface receptors, muscle structure and the immune system |

† Multiple domains in one transcript were counted only once.
