# Supplementary material for: Large-Scale Evidence for Conservation of NMD Candidature Across Mammals
Source: PLoS One. 2010 Jul 21;5(7):e11695. doi: 10.1371/journal.pone.0011695 (PMC2908137; doi:10.1371/journal.pone.0011695)
Supplement: Table S5 — Overrepresented KEGG pathways (0.04 MB DOC) [file pone.0011695.s006.doc]

| Table S5. Over-represented KEGG pathways |  |
| --- | --- |
| *Homo sapiens* | Corrected p-value |
| Glycine, serine and threonine metabolism (hsa00260) | 0.0109907 |
| Focal adhesion (hsa04510) | 0.0112022 |
| Epithelial cell signaling in Helicobacter pylori infection (hsa05120) | 0.0331909 |
| Polyunsaturated fatty acid biosynthesis (hsa01040) | 0.0344901 |
| Androgen and estrogen metabolism (hsa00150) | 0.0496874 |
| *Mus musculus* |  |
| ABC transporters (mmu02010) | 0.00927121 |
| Cell cycle (mmu04110) | 0.0097673 |
| mmu04115 | 0.0195789 |
| Polyunsaturated fatty acid biosynthesis (mmu01040) | 0.0373418 |
| RNA polymerase (mmu03020) | 0.0385474 |
| Oxidative phosphorylation (mmu00190) | 0.0423749 |
| mmu04120 | 0.0479475 |
| Carbon fixation (mmu00710) | 0.0520652 |
| Wnt signaling pathway (mmu04310) | 0.0523368 |
| Riboflavin metabolism (mmu00740) | 0.0831178 |
| *Rattus norvegicus* |  |
| Dorso-ventral axis formation (rno04320) | 0.0464317 |
| Bladder cancer (rno05219) | 0.0539997 |
| Endometrial cancer (rno05213) | 0.0615195 |
| Non-small cell lung cancer (rno05223) | 0.0764158 |
| Pancreatic cancer (rno05212) | 0.0764158 |
| *Bos taurus* |  |
| Ribosome (bta03010) | 0.00973015 |
| Colorectal cancer (bta05210) | 0.0152996 |
| Wnt signaling pathway (bta04310) | 0.0219541 |
| Prostate cancer (bta05215) | 0.0337998 |
| Adipocytokine signaling pathway (bta04920) | 0.0337998 |
| Basal cell carcinoma (bta05217) | 0.0401969 |
