# Supplementary material for: Large-Scale Evidence for Conservation of NMD Candidature Across Mammals
Source: PLoS One. 2010 Jul 21;5(7):e11695. doi: 10.1371/journal.pone.0011695 (PMC2908137; doi:10.1371/journal.pone.0011695)
Supplement: Table S6 — Overrepresented Biocarta pathways (0.03 MB DOC) [file pone.0011695.s007.doc]

| Table S6. Over-represented Biocarta pathways |  |
| --- | --- |
| *Homo sapiens* | Corrected p-value |
| h_mhcPathway | 0.0185305 |
| h_gleevecpathway | 0.0230575 |
| h_cell2cellPathway | 0.0262757 |
| h_integrinPathway | 0.031555 |
| h_ranMSpathway | 0.0461505 |
| *Mus musculus* |  |
| m_akap95Pathway | 0.0741685 |
| m_eifPathway | 0.0741685 |
| m_stathminPathway | 0.0897435 |
| m_tcytotoxicPathway | 0.0897435 |
| m_thelperPathway | 0.0897435 |
