# Supplementary material for: Large-Scale Evidence for Conservation of NMD Candidature Across Mammals
Source: PLoS One. 2010 Jul 21;5(7):e11695. doi: 10.1371/journal.pone.0011695 (PMC2908137; doi:10.1371/journal.pone.0011695)
Supplement: Table S7 — Overrepresented transcription factors (0.07 MB DOC) [file pone.0011695.s008.doc]

| Table S7. Over-represented transcription factors |  |
| --- | --- |
| *Homo sapiens* | Corrected p-values |
| E2F-1 | 2,71E-16 |
| Elk-1 | 9,01E-10 |
| MAZ | 1,03E-06 |
| LXR, PXR, CAR, COUP, RAR | 1,48E-05 |
| c-Ets-1p54 | 2,73E-05 |
| KROX | 2,34E-04 |
| TFII-I | 2,76E-05 |
| E2F | 3,52E-04 |
| USF | 7,00E-04 |
| E2F-1:DP-1 | 2,20E-02 |
| E2F-1:DP-2 | 1,83E-05 |
| E2F-4:DP-2 | 1,83E-05 |
| CREBATF | 5,40E-05 |
| NF-kappaB | 5,06E-05 |
| CP2 | 9,02E-05 |
| PITX2 | 8,13E-05 |
| Pax-3 | 9,01E-01 |
| Staf | 9,34734-e5 |
| c-Ets-1 68 | 7,58E-05 |
| CP2/LBP-1c/LSF | 0.000138788 |
| SREBP-1 | 0.000144244 |
| STATx | 0.000341622 |
| ATF3 | 0.00035843 |
| Sp3 | 0.000375979 |
| ATF-1 | 0.000466278 |
| ATF4 | 0.000556187 |
| Myc | 0.000902515 |
| CREB | 0.00105964 |
| NRF-2 | 0.00113819 |
| CRE-BP1:c-Jun | 0.00158674 |
| HES1 | 0.00153153 |
| Pax-9 | 0.00154919 |
| Rb:E2F-1:DP-1 | 0.00167123 |
| CRE-BP1 | 0.00179705 |
| c-Myc:Max | 0.00245067 |
| PPAR direct repeat 1 | 0.00256316 |
| Nkx2-5 | 0.00279448 |
| c-Myb | 0.00305903 |
| DEAF1 | 0.00341915 |
| Bach2 | 0.00387362 |
| Nrf-1 | 0.00404738 |
| MAZR | 0.00466424 |
| *Mus musculs* |  |
| PAX6 | 4,09E-30 |
| Major T-antigen | 2,42E-19 |
| Pax | 5,89E-10 |
| Imperfect Hogness/Goldberg BOX | 1,06E-09 |
| Elk-1 | 1,07E-07 |
| c-Ets-1p54 | 9,74E-06 |
| SREBP-1 | 2,81E-03 |
| TFII-I | 9,43E-02 |
| USF | 1,63E-01 |
| E2F-1 | 1,14E-01 |
| HEB | 1,05E-05 |
| Myc | 1,13E-05 |
| c-Ets-1 68 | 4,07E-05 |
| myogenin / NF-1 | 4,98E-04 |
| E2F | 8,73E-05 |
| SF-1 | 0.000311283 |
| Sp3 | 0.00082805 |
| TAL1 | 0.000854473 |
| NRF-2 | 0.00104817 |
| MyoD | 0.00168475 |
| Pax-3 | 0.00197134 |
| CREB | 0.0026648 |
| MAZR | 0.00263825 |
| ATF4 | 0.00364483 |
| CP2/LBP-1c/LSF | 0.00398967 |
| ER | 0.0055084 |
| PPAR direct repeat 1 | 0.00558692 |
| c-Myb | 0.00667532 |
| CP2 | 0.0127214 |
| CRE-BP1:c-Jun | 0.013007 |
| c-Myc:Max | 0.0124562 |
| Roaz | 0.0169657 |
| Brn-2 | 0.0190782 |
| c-Ets-1 | 0.0198571 |
| ATF3 | 0.0240781 |
| FOX | 0.0235538 |
| c-Ets-2 | 0.0232919 |
| NERF1a | 0.0252045 |
| MAZ | 0.0278705 |
| MAF | 0.0302364 |
| NF-E2 | 0.0335437 |
| YY1 | 0.0365219 |
| CREBATF | 0.0382342 |
| EBF | 0.0413336 |
| LXR, PXR, CAR, COUP, RAR | 0.0423288 |
| Staf | 0.048844 |
