# Supplementary material for: Large-Scale Evidence for Conservation of NMD Candidature Across Mammals
Source: PLoS One. 2010 Jul 21;5(7):e11695. doi: 10.1371/journal.pone.0011695 (PMC2908137; doi:10.1371/journal.pone.0011695)
Supplement: Table S8 — Classification of introns by size (0.03 MB DOC) [file pone.0011695.s009.doc]

| Intron length (Kb) | Non-retained Intron | Retained intron (R.I.) | Exon | R.I. conserved between human and mouse | R.I without match in PRIDE |
| --- | --- | --- | --- | --- | --- |
| < 0.1 | 51% | 55% | 55% | 0% | 52% |
| 0.1-0.5 | 51% | 58% | 58% | 61% | 57% |
| 0.5-1.0 | 47% | 57% | 62% | 59% | 56% |
| 1.0-1.5 | 46% | 54% | 59% | 56% | 53% |
| 1.5-2.0 | 44% | 54% | 59% | 57% | 56% |
| 2.0-2.5 | 44% | 52% | 47% | 57% | 54% |
| average % | 47% | 55% | 57% | 58% | 55% |

**Table S8: Classification of introns by size**
